# The Multi‐Functional Third Acceptor Realizes the Synergistic Improvement in Photovoltaic Parameters and the High‐Ratio Tolerance of Ternary Organic Photovoltaics

**DOI:** 10.1002/advs.202405303

**Published:** 2024-08-13

**Authors:** Yuhao Liu, Lingling Zhan, Zhongjie Li, Hang Jiang, Huayu Qiu, Xiaokang Sun, Hanlin Hu, Rui Sun, Jie Min, Jinyang Yu, Weifei Fu, Shouchun Yin, Hongzheng Chen

**Affiliations:** ^1^ Key Laboratory of Organosilicon Chemistry and Materials Technology of Ministry of Education College of Materials Chemistry and Chemical Engineering Hangzhou Normal University Hangzhou 311121 P. R. China; ^2^ Hoffmann Institute of Advanced Materials Shenzhen Polytechnic University Shenzhen 518055 P. R. China; ^3^ The Institute for Advanced Studies Wuhan University Wuhan 430072 P. R. China; ^4^ State Key Laboratory of Silicon and Advanced Semiconductor Materials Department of Polymer Science and Engineering Zhejiang University Hangzhou 310027 P. R. China

**Keywords:** high efficiency, high‐ratio tolerance, molecular design strategy, multi‐functional third acceptor, ternary organic photovoltaics

## Abstract

The ternary strategy proves effective for breakthroughs in organic photovoltaics (OPVs). Elevating three photovoltaic parameters synergistically, especially the proportion‐insensitive third component, is crucial for efficient ternary devices. This work introduces a molecular design strategy by comprehensively analyzing asymmetric end groups, side‐chain engineering, and halogenation to explore the outstanding optoelectronic properties of the proportion‐insensitive third component in efficient ternary systems. Three asymmetric non‐fullerene acceptors (BTP‐SA1, BTP‐SA2, and BTP‐SA3) are synthesized based on the Y6 framework and incorporated as the third component into the D18:Y6 binary system. BTP‐SA3, featuring asymmetric terminal (difluoro‐indone and dichloride‐cyanoindone terminal), with branched alkyl side chains, exhibited high open‐circuit voltage (*V*_OC_), balanced crystallinity and compatibility, achieving synergistic enhancements in *V*_OC_ (0.862 V), short circuit‐current density (*J*_SC_, 27.52 mA cm^−2^), fill fact (FF, 81.01%), and power convert efficiency (PCE, 19.19%). Device based on D18/Y6:BTP‐SA3 (layer‐by‐layer processed) reached a high efficiency of 19.36%, demonstrating a high tolerance for BTP‐SA3 (10–50%). This work provides novel insights into optimizing OPVs performances in multi‐component systems and designing components with enhanced tolerance.

## Graphical abstract

A molecular design strategy for the third component is proposed. Starting with the Y6 molecular framework, asymmetric non‐fullerene acceptors (BTP‐SA1, BTP‐SA2, BTP‐SA3) through end‐group, side‐chain, and halogenation modifications are created. The ternary device, D18/Y6:BTP‐SA3, shows a synergistic improvement in *V*_OC_, *J*_SC_, and FF, resulting in a high PCE of 19.36%, showcasing tolerance to varying component ratios (10–50%).

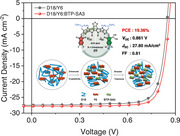

## 1 Introduction

Organic Photovoltaics (OPVs), which convert solar energy into electricity, hold significant promise for clean energy applications in the realm of renewable energy.^[^ ^1^, ^2^, ^3^ ^]^ Recent advancements in the design and development of efficient OPV materials, coupled with intelligent engineering optimizations, have led to rapid progress in OPVs.^[^ ^4^, ^5^, ^6^, ^7^, ^8^, ^9^, ^10^, ^11^, ^12^ ^]^ However, despite these strides, the power conversion efficiency (PCE) of OPV still lags behind their commercially established inorganic counterparts.^[^ ^13^, ^14^, ^15^ ^]^ The balance among three key performance parameters, open‐circuit voltage (*V*_OC_), short‐circuit current density (*J*_SC_), and fill factor (FF), remains a bottleneck. Additionally, energy loss (*E*_loss_), in comparison to inorganic solar cells, poses a significant hindrance to achieving efficiency breakthrough and industrial‐scale development.^[^ ^16^, ^17^ ^]^ Multi‐components strategy, involving energy level tuning, spectral complementarity, and morphology optimization, has proven to be effective approach for simultaneously enhancing the aforementioned performance parameters.^[^ ^18^, ^19^, ^20^, ^21^, ^22^, ^23^ ^]^ However, the sensitivity of the third component ratio has consistently limited the efficiency of high‐efficiency multi‐component systems.^[^ ^24^ ^]^ Therefore, the development of versatile third components that are insensitive to ratio variations, along with an exploration of energy loss mechanisms in multicomponent systems, is crucial for advancing the industrialization of OPVs.

In the realm of non‐fullerene acceptor material design, asymmetric end‐group construction with varying electron affinities has been explored to modulate the electronic energy level distribution, achieving a balance between *V*_OC_ and *J*_SC_.^[^ ^25^, ^26^, ^27^ ^]^ Intriguingly, certain asymmetric acceptors with dissimilar end‐groups have shown improved electroluminescent efficiency, leading to reduced *E*_loss_.^[^ ^28^, ^29^, ^30^ ^]^ Side‐chain engineering, particularly in the *β*‐side chains of Y6‐type systems, introduces bulky such as branched alkyl chains, effectively altering molecular stacking patterns.^[^ ^31^, ^32^, ^33^ ^]^ These molecules exhibit strong self‐aggregation and high crystallinity, with a diminishing impact on *π*–*π* stacking, resulting in a blue shift in the absorption spectrum and achieving a balance between *V*_OC_ and FF.^[^ ^34^ ^]^ Halogenation strategies, through end‐group halogenation, enable the modulation of molecular solubility, crystallinity, and compatibility with donor materials, optimizing morphology and achieving insensitivity to third‐component ratios, as well as a balance between *J*_SC_ and FF.^[^ ^35^, ^36^, ^37^, ^38^, ^39^ ^]^ In terms of *E*_loss_, aside from the inevitable radiative loss above bandgap, *E*_loss_ in OPVs primarily stems from the below bandgap radiative recombination loss and non‐radiative recombination loss. Both losses arise from the energy disorder in the system causing the recombination energy and low electroluminescent efficiency resulting from non‐radiative transitions, fundamentally occurring at the donor‐acceptor (D/A) interface.^[^ ^40^, ^41^, ^42^ ^]^ In the multi‐component system, the presence of additional interfaces complicates the *E*_loss_ analysis.^[^ ^43^, ^44^, ^45^, ^46^ ^]^ Thus, from the molecular structure design of the multi‐functional third component with broad proportion tolerance, to the control of *E*_loss_, we strive to identify the key factors balancing the three performance parameters and uncover the *E*_loss_ mechanisms in the multi‐component system to achieve the construction of efficient high‐performance multi‐component OPVs.

Based on the above considerations, we propose a comprehensive molecular design strategy that combines asymmetric end‐groups, side‐chain engineering, and halogenation strategies to explore outstanding optoelectronic properties of insensitive third components in efficient multi‐component OPVs. Utilizing the molecular framework of Y6, we substitute the IC end‐group with an asymmetric end‐group containing double chlorine substitutions (one end with bis‐chlorinated indene‐ketone and the other end with cyanide indene‐ketone) to produce the asymmetric non‐fullerene acceptor BTP‐SA1. This asymmetrical substitution reduces *E*_loss_, while the chlorine substitution broadens absorption. BTP‐SA1 is then incorporated as the third component into a D18:Y6 binary system to create a ternary device, achieving a balance between *V*_OC_ and *J*_SC_. Furthermore, we modify the alkyl side chain of BTP‐SA1 from a straight chain to a branched chain, leading to the formation of the asymmetric non‐fullerene acceptor BTP‐SA2. This substitution enhances *V*_OC_ and optimizes the molecular stacking and aggregation of the asymmetric acceptor, resulting in a ternary device that balances *V*_OC_ and FF. Finally, the bis‐chlorinated indene‐ketone end‐group of BTP‐SA2 was replaced with double fluorine substitutions. While maintaining a high *V*_OC_, adjusting molecular crystallinity and compatibility, achieving a high compatibility (insensitivity to component ratios), the asymmetric non‐fullerene acceptor BTP‐SA3 was prepared. In a ternary device, D18:Y6:BTP‐SA3 demonstrates a synergistic improvement in *V*_OC_ (0.862 V), *J*_SC_ (27.52 mA cm^−2^), and FF (81.01%), resulting in high‐efficiency (PCE = 19.19%). Additionally, to achieve greater insensitivity to ratios and efficiency improvement in ternary systems, a layer‐by‐layer (LbL) deposition method using the same solvent was adopted. The D18/Y6:BTP‐SA3 device achieves an efficiency enhancement of up to 19.36%, ranking among the highest efficiencies based on the D18:Y6 system. Moreover, the addition of BTP‐SA3 from 10% to 50% still maintains high photovoltaic performances, demonstrating excellent tolerance to the third component ratio. Our work provides new insights into the molecular design of additional functional components with high tolerance to component ratios, promoting the synergistic improvement of the three performance parameters in multi‐component OPVs, and offers a feasible pathway for the industrialization of it.

## 2 Results and Discussion

### 2.1 Synthesis and Characterization

**Figure 1:**
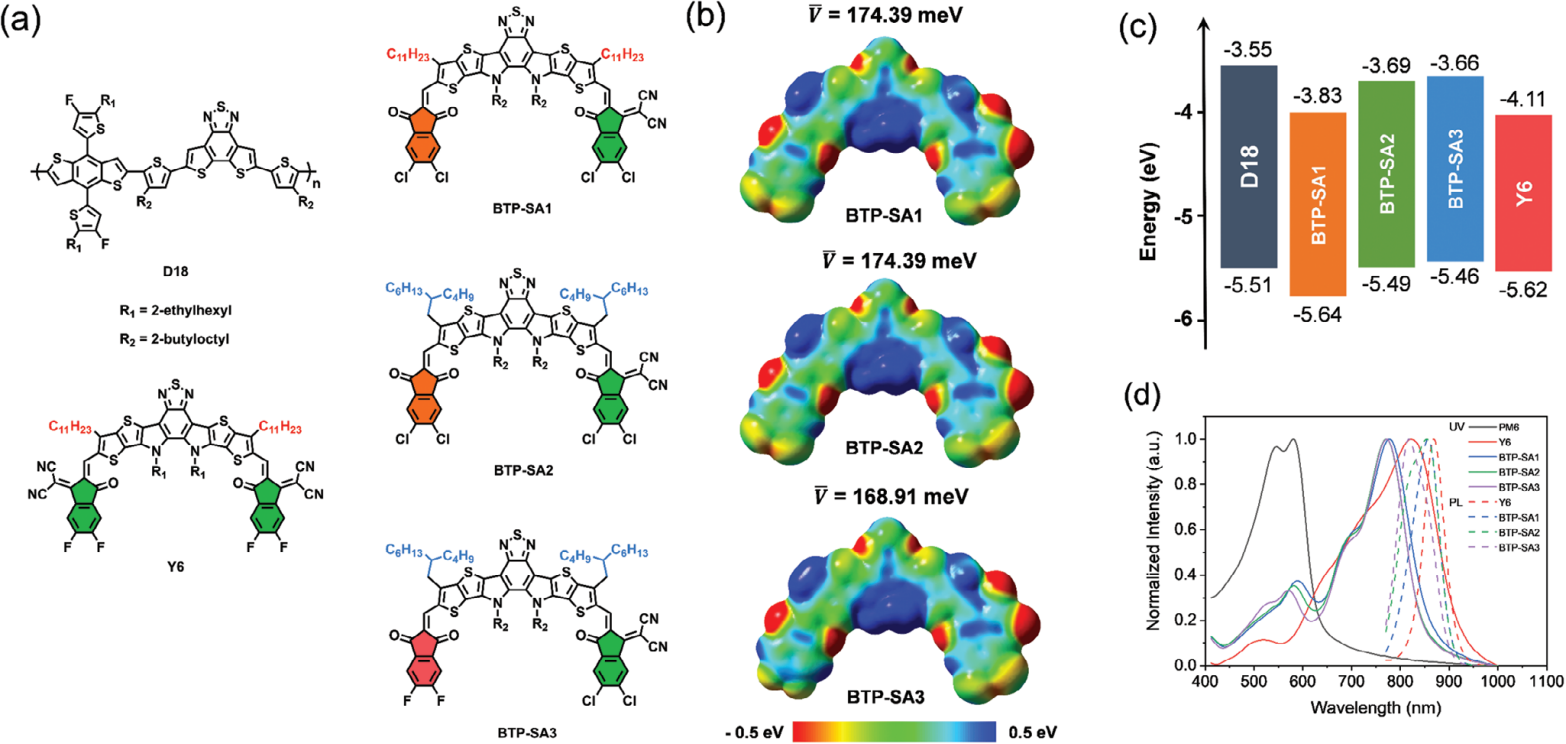
a) Chemical structures of D18, Y6, BTP‐SA1, BTP‐SA2, and BTP‐SA3. b) The electrostatic potential distributions of BTP‐SA1, BTP‐SA2, and BTP‐SA3. c) Energy diagram of corresponding materials determined by CV measurements. d) Normalized UV–vis and PL spectra of neat films.

Here, five OPV materials, namely D18, Y6, BTP‐SA1, BTP‐SA2, and BTP‐SA3 (refer to chemical structures in **Figure** **1**a) were investigated. D18 and Y6 are widely recognized molecules as the host binary system in this work,^[^ ^47^ ^]^ while the other three asymmetric non‐fullerene acceptors were newly synthesized through a one‐step Knoevenagel condensation reaction. Detailed information about their synthesis is available in the Supporting Information (Figures S1–S3, Supporting Information). The distinctions among these three electron acceptors primarily lie in their electron‐accepting terminals or alkyl side chains. Specifically, three terminals, namely 5,6‐dichloro‐1H‐indene‐1,3(2H)‐dione(IN‐2Cl), 2‐(5,6‐dichloro‐3‐oxo‐2,3‐dihydro‐1H‐inden‐1‐ylidene)malononitrile(IC‐2Cl), and 5,6‐difluoro‐1H‐indene‐1,3(2H)‐dione(IN‐2F), were selected for their distinct features in electron affinities and halogen types, potentially imparting unique characteristics to the relevant electron acceptors. Tuning alkyl side chains between BTP‐SA1 and BTP‐SA2 may impact molecular packing, while the alternation of halogen types between BTP‐SA2 and BTP‐SA3 could influence miscibility properties, thereby leading to varied photovoltaic performances. Thus, the effects of molecular symmetry, alkyl side chains, and terminal groups on parameters such as *E*_loss_, morphological characteristics, macroscopic properties of active layers, and optoelectronic properties of OPVs were systematically studied. This comprehensive analysis aims to derive meaningful molecular structure‐performance relationships.

To calculated the molecular electrostatic potentials (ESPs), density functional theory (DFT) calculations were performed (the simplified calculation is model the side chains as methyl groups) and the results were presented in Figure 1b and Figure S4 (Supporting Information). It was found that with alternation from IN‐2Cl to IN‐2F, BTP‐SA3 was shown a lower ESPs, thus possessing a weaker intermolecular interaction. To compare the energy levels of these acceptors, cyclic voltammetry (CV) method was applied (Figure 1c; Figure S5, Supporting Information). As expected, the three new asymmetric non‐fullerene acceptors owned the lowest unoccupied molecular orbital (LUMO) level of −3.83, −3.69, and −3.66 eV, the highest occupied molecular orbital (HOMO) level of −5.64, −5.49, and −5.46 eV, for BTP‐SA1, BTP‐SA2 and BTP‐SA3, respectively. As the third component (acceptors), the higher LUMO level could be beneficial for enhancing *V*_OC_ in the ternary system.

Figure 1d shows the optical properties of five OPV materials, characterized by UV–vis absorption and steady‐state fluorescence (PL) spectra. The rational complemented absorption covering 400–1000 nm from the visible to near‐infrared region provides the high‐efficiency potential of the ternary systems. In addition, PL of BTP‐SA series overlaps with the absorption spectrum of Y6 to a large extent, which can reuse the photo‐energy to improve the photo‐current response of the ternary devices.^[^ ^48^ ^]^

### 2.2 Miscibility and BHJ‐Film Morphology

**Figure 2:**
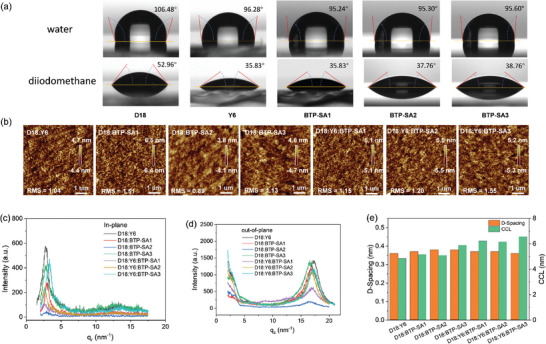
a) Contact angle images of various films with water and diiodomethane droplets on top. b) AFM height images of four binary (D18:Y6, D18:BTP‐SA1, D18:BTP‐SA2, and D18:BTP‐SA3) and three ternary (D18:Y6:BTP‐SA1, D18:Y6:BTP‐SA2, and D18:Y6:BTP‐SA3) blend films. Intensity profiles along c) the in‐plane, and d) out‐of‐plane directions. e) D‐spacing and CCL values of different blend films.

To assess the miscibility as indicated by the Flory–Huggins interaction parameter (*χ*_D‐A_) and the multi‐phase morphology suggested by the wetting coefficient (*ω*) in the ternary blend, contact angle measurements with water and diiodomethane were conducted on both neat and blended films (refer to **Figure** **2**a; Figure S6, Supporting Information). *χ*_D‐A_ (Table S1, Supporting Information) presents the miscibility between two materials, and the smaller *χ*_D‐A_ means more miscibility.^[^ ^49^ ^]^ The value of *χ*_D‐A_ is calculated as 0.28, 0.49, 0.39, and 0.32, between the donor (D18) and acceptors (Y6, BTP‐SA1, BTP‐SA2 and BTP‐SA3), respectively. Y6 shows highest miscibility with D18 than that of BTP‐SA series. Notably, introducing less compatible asymmetric acceptors into the D18:Y6 system can modulate the compatibility between D and A, which may inhibit previously excessively small phase separation, and result in the appropriate domain size. Furthermore, *ω*, which infers the location of the third component in the host blend, was calculated (Table S1, Supporting Information). If *ω*_A2_ is smaller than −1, the third acceptor (A2) will infuse into the phase of the host acceptor (A1), if *ω*_A2_ is larger than 1 while into the domain of the host donor (D), and if −1 < *ω*_A2_ < 1, A2 will be located at the interfaces of D and A1.^[^ ^50^ ^]^ For BTP‐SA1 based ternary blend, the third component is merged into Y6 to form the well‐mixed acceptor domains (*ω*_A2_ is −1.09).^[^ ^45^ ^]^ Due to the minimal compatibility between BTP‐SA1 and D18, it exhibits a higher degree of mixing with the Y6 phase in the heterojunction. For the BTP‐SA2 and BTP‐SA3‐based ternary blend, the third component is positioned at the interface between D18 and Y6 (*ω*_A2_ is −0.84 and −0.86, respectively), resulting a cascade‐type junction, which can enhance the phase purity of the donor and facilitate additional exciton dissociation interfaces and charge transport channels.^[^ ^20^, ^51^ ^]^

The surface morphology and aggregation properties of neat (Figure S7, Supporting Information), binary and ternary blend films (Figure 2b) were investigated using atomic force microscopy (AFM). As depicted in Figure S7 (Supporting Information), D18 exhibits pronounced crystallinity and cluster‐like surface morphology, with a root mean square (RMS) roughness of 1.36 nm. Y6 and BTP‐SA1, featuring straight‐chain alkyl side chains, display relatively smaller surface roughness with RMS distributions of 1.05 and 0.92 nm, respectively. In contrast, BTP‐SA2, characterized by branched alkyl side chains terminated with chlorine, demonstrates the highest surface roughness (RMS = 1.24 nm). However, upon replacing the terminal chlorine with fluorine, BTP‐SA3 exhibits a suitable roughness (RMS = 1.10 nm). After blended BTP‐SA series materials into host binary D18:Y6 system, the ternary blend films have their own morphology properties. Compared to the BTP‐SA2 and BTP‐SA3 based ternary blends, BTP‐SA1‐based ternary blend show well‐mixed acceptor domain, thus resulting in a lower roughness (RMS = 1.15 nm). For BTP‐SA2 and BTP‐SA3‐based ternary films significant cascade‐type morphology are formed, which exhibits the larger roughness. From the phase diagram results (Figure S8, Supporting Information), a finer phase separation morphology exists for the ternary system of BTP‐SA3‐based, which offers the possibility of balancing the photocurrent and FF of its devices.

Grazing incidence wide‐angle X‐ray scattering (GIWAXS) characterization was performed to explore the orientation, packing, and crystallinity of molecules in the neat and blend films.^[^ ^52^ ^]^ The 2D GIWAXS patterns, and the corresponding 1D profiles along out‐of‐plane (OOP) and in‐plane (IP) directions of neat films (Figure S9, Supporting Information), four binary blends, and three ternary blends (Figure 2c,d; Figure S10, Supporting Information) are shown. The molecular spacing distance (D‐spacing) and crystallization coherence length (CCL) is quantified as depicted in Figure 2e, and relevant parameters are listed in Table S2 (Supporting Information). In the pure film, OOP direction, at the *π*–*π* stacking peaks, BTP‐SA2 and BTP‐SA3 show strong face‐on orientation, attributed to the orderly arrangement of molecules. This is consistent with literature reports, indicating that side chains with branched alkyl moieties contribute to more orderly molecular stacking and show strong crystallinity, facilitating charge transport in the vertical direction.^[^ ^32^, ^34^ ^]^ Thus, diffraction peaks for BTP‐SA2 and BTP‐SA3 are stronger than those of BTP‐SA1 in both IP and OOP directions. However, having four chlorine atoms in the terminal substitute, BTP‐SA2 possesses high self‐aggregation tendency, which may affect the molecular packing in bulk heterojunction (BHJ) blend, thus influencing the crystallinity of BTP‐SA2‐based blend film. In comparison to BTP‐SA2, the fluorine substitution in BTP‐SA3 leads to a reduction in electrostatic potential, thereby diminishing the interaction forces between host and guest molecules. With the introduction of the asymmetric acceptor BTP‐SA3 into the D18:Y6 blend, a suitable morphology with high crystallinity of donor and acceptor within the active layer are maintained. Notably, the original binary system D18:Y6 has the lowest CCL, and with the addition of the asymmetric acceptor, D‐spacing kept and CCL values significantly increase. Among them, the BTP‐SA3 system shows the largest CCL increase, consistent with charge mobility results. All these results might contribute to the enhancement and balance of *J*_SC_ and FF in the BTP‐SA3‐based ternary system.

### 2.3 Photovoltaic Performance

**Figure 3:**
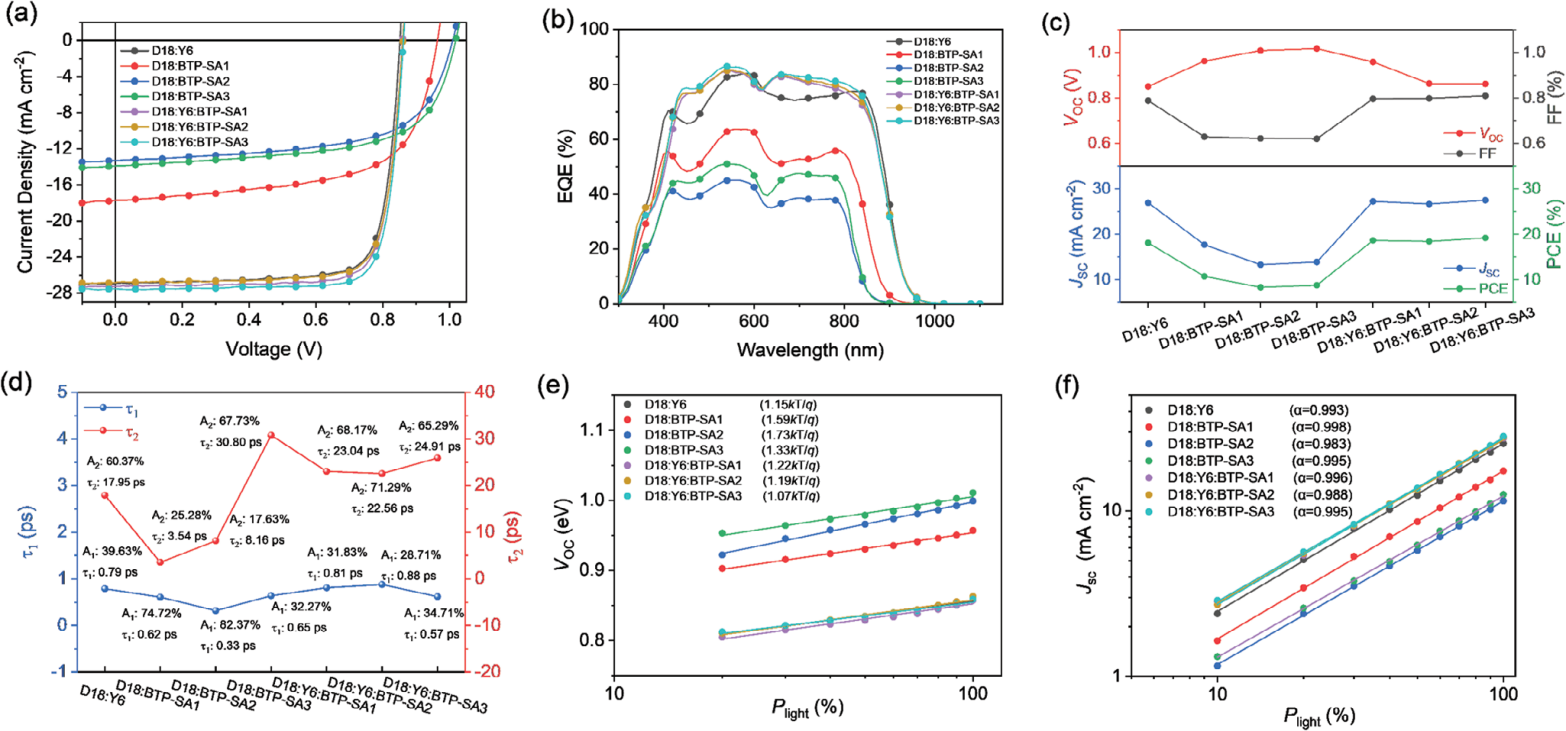
a) *J*–*V* curves, b) EQE curves and c) photovoltaics parameters variations of four binary (D18:Y6, D18:BTP‐SA1, D18:BTP‐SA2, and D18:BTP‐SA3) devices and three ternary (D18:Y6:BTP‐SA1, D18:Y6:BTP‐SA2, and D18:Y6:BTP‐SA3) devices. d) Comparisons of τ_1_ and τ_2_ of different blends. e) *V*_OC_ and f) *J*_SC_ on light intensity of the corresponding OPVs.

To evaluate the impact of the newly synthesized asymmetric non‐fullerene acceptors on the photovoltaic performances in ternary devices, the devices based on four binary and three ternary systems were fabricated with a conventional structure (ITO/PEDOT:PSS/active layer/PDINN/Ag). The detailed devices fabrication procedures could be found in Electronic Supplementary Information. The current‐density–voltage (*J*–*V*) curves of the devices under AM 1.5G 100 mW cm^−2^ illumination (SS‐F5‐3A, Enlitech) are shown in **Figure** **3**a and the corresponding photovoltaic parameters are summarized in **Table** **1**. The device based on D18:Y6 displays a feasible PCE of 18.10%, with a *V*_OC_ of 0.851 V, a *J*_SC_ of 26.94 mA cm^−2^, and an FF of 78.89%, which is corresponding to the reported values. Compared with Y6 based binary device, BTP‐SA1–A3 based binary device possesses the higher *V*_OC_ of 0.963–1.018 V as expected. With the addition of weight ratio of 10% BTP‐SA1 into the D18:Y6 blend, the ternary device shows PCE as high as 18.65% with synergistically an enhanced *V*_OC_ of 0.859 V and an enhanced *J*_SC_ of 27.27 mA cm^−2^, and a comparable FF of 78.90%. With the addition of BTP‐SA2 (with the same added weight ratio of 10%) into the host binary, the ternary device achieved a high PCE of 18.43%, both improved in *V*_OC_ (0.864 V) and FF (79.88%), and a suitable *J*_SC_ of 26.68 mA cm^−2^. While for BTP‐SA3 based ternary device, all the photovoltaic performances have been boosted, with a high *V*_OC_ of 0.862 V, a surprised *J*_SC_ of 27.52 mA cm^−2^, and a desirable FF of 81.01%, resulting in an outstanding PCE of 19.19%, which is one of the highest efficiencies among the Y6‐based OPVs. The reliability of the measured device performance could be confirmed by the reasonable error of *J*_SC_ between the values calculated from the external quantum efficiencies (EQEs, Figure 3b) and the values measured by *J*–*V* curves, thus verifying the repeatability of our work. The device performances with detailed photovoltaic parameters and variation trends in the different binary and ternary blends are depicted in Figure 3c. Histograms of PCE values of various binary and ternary devices are displayed in Figure S11 (Supporting Information).

**Table 1:** The photovoltaic parameters of the devices based on different acceptor components.

Active Layer	*V*_oc_ [V]	*J*_sc_ [mA cm^−2^]	*J*_cal_ ^a)^ [mA cm^−2^]	FF [%]	PCE _b)_ [%]
D18:Y6	0.851 (0.855 ± 0.002)	26.94 (26.21 ± 0.36)	25.06	78.89 (79.20 ± 1.14)	18.10 (17.76 ± 0.17)
D18:BTP‐SA1	0.963 (0.958 ± 0.005)	17.70 (17.17 ± 0.36)	16.69	62.93 (62.85 ± 0.64)	10.73 (10.35 ± 0.24)
D18:BTP‐SA2	1.009 (1.009± 0.003)	13.30 (13.20 ± 0.62)	11.20	62.19 (59.79 ±1.81)	8.32 (7.94 ± 0.54)
D18:BTP‐SA3	1.018 (1.010 ± 0.005)	13.88 (13.22 ± 0.41)	13.05	62.06 (62.14 ± 0.57)	8.76 (8.28 ± 0.26)
D18:Y6:BTP‐SA1	0.859 (0.858± 0.003)	27.27 (27.04 ± 0.15)	25.64	79.67 (78.99 ± 0.65)	18.65 (18.30 ± 0.18)
D18:Y6:BTP‐SA2	0.864 (0.863 ± 0.002)	26.68 (26.74 ± 0.20)	25.93	79.88 (79.69 ± 0.28)	18.43 (18.41 ± 0.12)
D18:Y6:BTP‐SA3	0.862 (0.861 ± 0.003)	27.52 (27.09 ± 0.40)	26.21	81.01 (80.54 ± 0.52)	19.19 (18.78 ± 0.36)

^a)^ Integrated current densities from EQE curves;

^b)^ Average PCEs from ten devices.

To investigate the tolerance of BTP‐SA series molecules in mixed heterojunctions, ternary devices with varying contents of the third component were fabricated. As shown in Figure S12 and Table S3 (Supporting Information), with the increasing addition of BTP‐SA series molecules (wt.% from 10% to 50%), the ternary devices exhibit the reasonable enhancement in *V*_OC_. However, with the decrease in miscibility between BTP‐SA series molecules and D18, a significant performance decline is observed in the ternary systems based on the BTP‐SA series. Notably, among the molecules studied, the ternary system based on BTP‐SA3 demonstrates the best doping tolerance compared to that base on the other two molecules, which would further confirm in LbL‐type devices.

### 2.4 Exciton and Charge Behaviors

Transient absorption spectroscopy (TAS) was conducted to study the exciton separation, charge‐transfer kinetics of these OPVs. Corresponding TA spectra of the neat acceptor films and binary and ternary blended films are depicted as Figures S13 and S14 (Supporting Information), and the hole transfer kinetics were fitted and extracted (relevant data were listed in Table S4, Supporting Information).^[^ ^53^ ^]^ As noted in Figure 3d, the time constant τ_1_ represents the dissociation time of excitons at the D/A interface, while τ_2_ represents the time it takes for excitons to diffuse to the interface. It could be observed that in the D18:Y6 and D18:BTP‐SA3 films, the dissociation time of excitons at the interface is similar (τ_1_ is 0.79 ps for D18:Y6 blend, and is 0.65 ps for D18:BTP‐SA3 blend), but the time taken for excitons to diffuse to the interface (τ_2_ is 17.95 ps for D18:Y6 blend, and is 30.80 ps for D18:BTP‐SA3 blend) is nearly doubled in the BTP‐SA3 based binary blend. This is attributed to the comparable D‐A compatibility and blend roughness in these two binary systems, but with BTP‐SA3 exhibiting a larger CCL value, necessitating a longer diffusion time. For ternary blend, with the addition of BTP‐SA1 and BTP‐SA2 into the host binary, the significant crystallinity reduction could be found, thus influence the exciton separation in the interface of D/A, and resulting the lower τ_1_ (τ_1_ is 0.79, 0.81, and 0.88 ps, for the D18:Y6, D18:Y6:BTP‐SA1 and D18:Y6:BTP‐SA2 blend films, respectively). With the introduction of asymmetric acceptors, the diffusion time of excitons in ternary systems is increased (τ_2_ is 23.04, 22.56, and 24.91 ps, for the D18:Y6:BTP‐SA1–A3 blend, respectively), stemming from enhanced CCL values. As expected, the ternary systems based on BTP‐SA3, the shortest exciton dissociation time is observed (τ_1_ = 0.57 ps), owing to its refined D‐A phase separation dimensions and high crystallinity. Consequently, ternary systems based on BTP‐SA3 possesses ultrafast exciton dissociation and prolonged exciton transport time, facilitating a synergistic enhancement in *J*_SC_ and FF.

*J*–*V* curves under different light intensities (*P*_light_) were conducted on devices based on various binary and ternary systems to study the charge recombination situations. The monomolecular recombination could be described through the relationship between *V*_OC_ and *P*_light_ (n*k*T/*q*, *k* is the Boltzmann constant, T is the absolute temperature, *q* is the elementary charge). As demonstrated in Figure 3e, the ternary system based on BTP‐SA3 demonstrates the minimal degree of monomolecular recombination (1.07*k*T/*q*), contribution to the highest FF. Similarly, the bimolecular recombination could be estimated by employing the relation of *J*_SC_ ∼ *P*_light_ *^α^* (Figure 3f).^[^ ^54^, ^55^ ^]^ Again, the ternary device based on BTP‐SA3 exhibits lower bimolecular recombination (*α*  = 0.995), also facilitates to its high FF.

The space‐charge limited current (SCLC) method was applied to estimate the charge mobilities of various blends. The ternary D18:Y6:BTP‐SA1 and D18:Y6:BTP‐SA3 blends exhibit the higher hole and electron mobilities (*µ*_h_ is 7.31 × 10^−4^ cm^2^ V^−1^ s^−1^, 7.79 × 10^−4^ cm^2^ V^−1^ s^−1^, *µ*_e_ is 4.28 × 10^−4^ cm^2^ V^−1^ s^−1^, 7.00 × 10^−4^ cm^2^ V^−1^ s^−1^, for BTP‐SA1‐based, BTP‐SA3‐based ternary blend, respectively) than that of host binary blends (*µ*_h_ is 6.04 × 10^−4^ cm^2^ V^−1^ s^−1^, *µ*_e_ is 3.81 × 10^−4^ cm^2^ V^−1^ s^−1^), as shown in Figure S15 and Table S5 (Supporting Information). A significant enhancement in both *µ*_h_ and *µ*_e_ may be one of the reasons for the higher *J*_SC_ of the BTP‐SA1 and BTP‐SA3 based ternary devices. For BTP‐SA2 based ternary blend, the lower charge mobility (*µ*_h_ is 4.98 × 10^−4^ cm^2^ V^−1^ s^−1^, *µ*_e_ is 3.62 × 10^−4^ cm^2^ V^−1^ s^−1^) consisted with the lower stacking intensity in face‐on direction and severe bimolecular recombination, thus influencing the *J*_SC_ for BTP‐SA2 based devices.

### 2.5 Energy Loss

**Figure 4:**
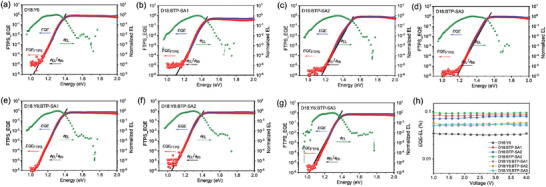
Semilogarithmic plots of normalized EL spectra, measured EQE spectra, and FTPS‐EQE spectra as a function of energy for devices based on a) D18:Y6, b) D18:BTP‐SA1, c) D18:BTP‐SA2, d) D18:BTP‐SA3, e) D18:Y6:BTP‐SA1, f) D18:Y6:BTP‐SA2, and g) D18:Y6:BTP‐SA3 device. h) EQE‐EL of OPVs with different components at various bias voltages.

To explain the variation in voltage for ternary devices, a detailed analysis of *E*_loss_ for the binary and ternary OPVs was carried out.^[^ ^56^ ^]^ The quantified formula of *E*_loss_ could be found in the Supporting Information. The characterization method for calculating *E*_loss_ is shown in **Figure** **4**, and the calculated detailed *E*_loss_ is shown in **Table** **2**. It's found that with the asymmetric end‐groups *E*_loss_ of three asymmetric acceptors are both small (*E*_loss_ is 0.533 eV for D18:Y6, 0.496 eV for D18:BTP‐SA1, 0.514 eV for D18:BTP‐SA2, and 0.498 eV for D18:BTP‐SA3), especially in reduction of the Δ*E*_3_ part, thus enabling a higher *V*_OC_ for ternary devices than host binary OPV, and verifying asymmetric acceptors as a low‐energy‐loss material as the intended design strategy.

**Table 2:** Detailed energy losses of OPVs based on various binary and ternary devices.

Active layers	*E*_loss_ [eV]	$\Delta E_{1}=E_{gap}-qV_{oc}^{SQ}$ [eV]	$\Delta E_{2}=\;q\Delta V_{oc}^{rad}$ [eV]	$\Delta E_{3}=q\Delta V_{oc}^{non-rad}$ [eV]	EQE_EL_ [%]	$\mathrm{Exp}.\;q\Delta V_{oc}^{non-rad}\;[\mathrm{eV}]$
D18:Y6	0.533	0.252	0.081	0.198	3.80E‐2	0.207
D18:BTP‐SA1	0.496	0.258	0.061	0.177	8.38E‐2	0.183
D18:BTP‐SA2	0.514	0.262	0.065	0.186	7.57E‐2	0.186
D18:BTP‐SA3	0.498	0.264	0.056	0.178	9.28E‐2	0.181
D18:Y6:BTP‐SA1	0.527	0.251	0.081	0.194	5.16E‐2	0.196
D18:Y6:BTP‐SA2	0.523	0.251	0.079	0.192	5.50E‐2	0.194
D18:Y6:BTP‐SA3	0.525	0.251	0.082	0.192	5.37E‐2	0.194

The term Δ*E*_3_ is intimately connected to the luminescence efficiency of photovoltaic materials, as described by the equation:

(1) $$\Delta \;E_{3}=q\Delta \;V_{\mathrm{oc}}^{\mathrm{non}-\mathrm{rad}}=\;-kT\ln\left(\mathrm{EQ}E_{\mathrm{EL}}\right)$$

wherein *k* is the Boltzmann constant, *T* is the temperature in Kelvin, EQE_EL_ is the electroluminescence quantum efficiency of the device. As shown in Figure 4h and Table 2, the D18:BTP‐SA3 device has the highest EQE_EL_ of 9.28 × 10^−2^%. As the asymmetric acceptors is added as the third‐component, ternary devices achieved much higher EQE_EL_ (5.16 × 10^−2^% for D18:Y6:BTP‐SA1, 5.50 × 10^−2^% for D18:Y6:BTP‐SA2, 5.37 × 10^−2^% for D18:Y6:BTP‐SA3) than that of host binary device (3.80 × 10^−2^%). The enhanced luminescence efficiency results in a reduction in Δ*E*_3_. Fluorescence spectroscopy tests (Figure S16, Supporting Information) on diverse acceptor mixtures confirmed that the addition of high‐luminance acceptors, the BTP‐SA series, significantly enhances the emission efficiency of the host‐guest acceptor film. The above results indicate that the enhanced luminescence efficiency of ternary devices is derived from the high luminescence efficiency of the BTP‐SA1–3, which is in accordance with the EL results and could explain well the reduced non‐radiative recombination loss (Δ*E*_3_) in the ternary device,^[^ ^26^, ^43^ ^]^ thus enabling an increased voltage.

### 2.6 Layer‐by‐Layer Processed Devices with High‐Ratio Tolerance

**Figure 5:**
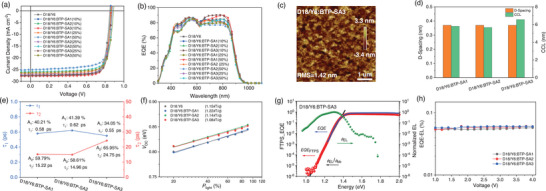
a) *J*–*V* curve, and b) EQE curve of LbL‐type device based on host binary (D18/Y6) and three ternary systems (D18/Y6:BTP‐SA1, D18/Y6:BTP‐SA2, and D18/Y6:BTP‐SA3). c) AFM height images of D18/Y6:BTP‐SA3 blend. d) D‐spacing and CCL values of three LbL‐type ternary blends. e) Comparisons of τ_1_ and τ_2_ of three LbL‐type ternary blends. f) Dependence of *V*_OC_ on light intensity of the LbL‐type ternary devices. g) Semilogarithmic plots of normalized EL spectra, measured EQE spectra, and FTPS‐EQE spectra as a function of energy for devices based on BTP‐SA3‐based LbL‐type ternary film. h) EQE_EL_ of OPVs based on three LbL‐type ternary systems at various bias voltages.

The LbL deposition method has been demonstrated as an effective approach to enhance vertical charge transport and achieve performance breakthroughs in OPVs, even neglecting the D:A ratio.^[^ ^57^, ^58^ ^]^ In ternary systems, the proportion of the third component in mixed‐heterojunction blends is typically sensitive, posing a challenge for achieving high proportions of the third component doping. In our previous work, it was found that employing the LbL method with the same solvent could realize a better interpenetrating network structure for D and A in the vertical direction, further achieving efficiency breakthroughs and providing the possibility for high proportion third‐component doping.^[^ ^59^ ^]^ In this work, we employed the LbL method with the same solvent to fabricate ternary systems with proportion‐insensitive characteristics. Based on the optimized A1:A2 ratio of the BHJ‐type ternary system, the prepared LbL‐type devices exhibited improvements in *V*_OC_, *J*_SC_, and FF, ultimately demonstrating higher efficiencies (**Figure** **5**a and **Table** **3**). Based on BTP‐SA3, LbL‐type ternary devices, an obvious increased efficiency of 19.36% was achieved, which stands as one of the highest efficiencies for Y6‐based OPVs. Furthermore, we conducted the device fabrication with high‐ratio doping of the third component of wt.% from 10% to 50%. With the addition of BTP‐A1 from 25% to 50% weight ratio, except the *V*_OC_, the *J*_SC_, the FF and the PCE are all reduced with evident decline. For BTP‐SA2‐based ternary devices, *V*_OC_ increased with expected, but *J*_SC_ and FF decreased obviously, PCE is 18.8% for 25% BTP‐SA2 based LbL‐type ternary device, and reduced to 17.6% for 50% BTP‐SA2‐based LbL‐type ternary one. While for BTP‐SA3 based LbL‐type ternary OPV, 25% weight ratio device shows a high *V*_OC_ of 0.871 V, and maintains relatively high *J*_SC_ and FF, resulting in a desired efficiency as high as 19.23%. Even with the high‐ratio of 50%, the ternary device based on BTP‐SA3 could also maintain the high efficiency over 18%, confirming that the newly designed asymmetric acceptor BTP‐SA3, with considerations for crystallinity and compatibility, could realize the synergistic improvement of various photovoltaic parameters and the high proportion tolerance of ternary OPVs.

**Table 3:** The performance parameters of the device based on the LbL‐type devices.

Active Layer	*V*_oc_ [V]	*J*_sc_ [mA cm^−2^]	*J*_cal_ ^a)^[mA cm^−2^]	FF [%]	PCE ^b)^ [%]
D18/Y6	0.849 (0.844 ± 0.004)	27.47 (27.26 ± 0.31)	26.87	79.17 (79.48 ± 0.45)	18.48 (18.29 ± 0.23)
D18/Y6:BTP‐SA1 (wt.%:10%)	0.855 (0.855 ± 0.002)	27.66 (27.37 ± 0.28)	26.36	80.93 (80.13 ± 0.59)	19.05 (18.90 ± 0.08)
D18/Y6:BTP‐SA2 (wt.%:10%)	0.859 (0.859 ± 0.001)	27.61 (27.35 ± 0.30)	26.57	80.02 (79.90 ± 0.29)	18.93 (18.85 ± 0.05)
D18/Y6:BTP‐SA3 (wt.%:10%)	0.861 (0.860 ± 0.003)	27.80 (27.38 ± 0.32)	26.77	80.95 (80.54 ± 0.56)	19.36 (18.93 ± 0.25)
D18/Y6:BTP‐SA1 (wt.%:25%)	0.863 (0.866 ± 0.003)	26.81 (24.72 ± 0.30)	26.01	78.12 (77.93 ± 0.46)	18.05 (17.48 ± 0.48)
D18/Y6:BTP‐SA1 (wt.%:50%)	0.879 (0.876 ± 0.003)	25.03 (24.25 ± 0.40)	24.87	74.78 (76.40 ± 0.95)	16.45 (16.23 ± 0.34)
D18/Y6:BTP‐SA2 (wt.%:25%)	0.871 (0.870 ± 0.004)	26.97 (27.15 ± 0.24)	25.67	79.95 (79.56 ± 0.55)	18.80 (18.74 ± 0.04)
D18/Y6:BTP‐SA2 (wt.%:50%)	0.889 (0.884 ± 0.002)	25.90 (25.99 ± 0.11)	25.75	76.59 (76.33 ± 0.31)	17.64 (17.62 ± 0.06)
D18/Y6:BTP‐SA3 (wt.%:25%)	0.871 (0.871 ± 0.001)	27.54 (27.31 ± 0.31)	27.36	80.24 (79.90 ± 0.29)	19.23 (19.01 ± 0.12)
D18/Y6:BTP‐SA3 (wt.%:50%)	0.889 (0.885 ± 0.002)	26.26 (26.09 ± 0.15)	25.22	78.53 (78.03 ± 0.46)	18.32 (18.00 ± 0.16)

^a)^ Integrated current densities from EQE curves;

^b)^ Average PCEs from ten devices.

The reliability of the measured device performance could be confirmed by the reasonable error of *J*_SC_ between the values calculated from the EQEs (Figure 5b) and the values measured by *J*–*V* curves. For the ternary system prepared by the LbL method, comprehensive analyses including film surface morphology, molecular stacking orientation within the active layer, coherence length of crystalline phases, behaviors of excitons and charge carriers, energy losses analysis were also conducted. As depicted in Figure S17 (Supporting Information), due to the formation a well‐mixed phase between BTP‐SA1 and Y6, films prepared by stepwise deposition exhibited minimal surface roughness with an RMS of 1.02 nm. Lacking the bondage of D18 donor interpenetrating network framework, BTP‐SA2, with branched alkyl side‐chains and bis‐chloro substituents, possesses strong self‐aggregation property, resulting in the highest surface roughness (RMS = 3.16 nm) in the LbL‐type blend. While, BTP‐SA3, possessing balanced crystallinity and compatibility, showed appropriate surface roughness with a suitable RMS of 1.42 nm (Figure 5c). GIWAXS results were consistent with AFM results, indicating that the BTP‐SA2 based ternary system exhibited the strongest crystallinity with the highest diffraction peak intensity in all directions, as shown in Figure S18 (Supporting Information). While BTP‐SA3 based LbL‐type ternary device demonstrated a larger CCL value (Figure 5d; Table S6, Supporting Information, CCL for D18/Y6:BTP‐SA3 is 6.57 nm, which is the highest among the three LbL‐type ternary blends) and suitable crystallinity, leading to synergistic enhancements in *J*_SC_ and FF. As demonstrated in Figure 5e (hole transfer kinetic was fitted and extracted from the TAS spectra, Figure S19, Supporting Information), LbL‐type ternary devices fabricated based on BTP‐SA3 exhibited the fastest exciton interface dissociation time (τ_1_ = 0.55 ps), longer exciton migration lifetime (τ_2_ = 24.75 ps, Table S7, Supporting Information), and reduced charge recombination (Figure 5f and n = 1.08; Figure S20, Supporting Information, *α* = 0.996), highest electron mobility (Figure S21 and Table S8, Supporting Information, *µ*_e_ = 4.44 × 10^−4^ cm^2^ V^−1^ s^−1^), consistent with the optimized film morphology and largest CCL value of BTP‐SA3. As for *E*_loss_ aspect, all LbL‐type ternary devices show the reduced *E*_loss_ (Figure S22 and Table S9, Supporting Information), resulting in the higher *V*_OC_. Especially, BTP‐SA3 based ternary system shows the lowest non‐radiative recombination losses (Figure 5g; Table S9, Supporting Information, Δ*E*_3_ = 0.189 V), which might be originated from the higher emission intensity (confirmed in Figure S16, Supporting Information), and the highest electroluminescence efficiency (Figure 5h, EQE_EL_ = 5.94×10^−2^%). Considering these factors collectively, the ternary devices based on BTP‐SA3 demonstrated simultaneous improvements in photovoltaic performance parameters and optimal device efficiency, and high‐ratio tolerance.

## 3 Conclusion

In summary, the desired multi‐functional third component was designed and synthesized, and high‐efficiency ternary OPV with synergistic enhancements in three photovoltaic performances and broad addition‐ratio was achieved in this work. Benefit from the asymmetric molecular configuration, branched alkyl side‐chain substitutions, and fluorine‐chlorine turning terminal groups strategies, BTP‐SA3 possesses excellent excitonic and charge carrier behaviors, low energy losses, and balanced crystallinity and compatibility, and high‐ratio tolerance with the main binary system. Thus, the BHJ‐type ternary device (D18:Y6:BTP‐SA3, wt.% = 10%) shows an efficiency of 19.19%. The LbL‐type ternary device (D18/Y6:BTP‐SA3, wt.% = 10%) processed with the same chloroform solvent exhibits an excellent photovoltaic performance with a PCE of 19.36%, which is one of the highest values among previously reported Y6‐based OPVs. Furthermore, with a high‐ratio (wt.% = 50%) of BTP‐SA3 based LbL‐type ternary device, it exhibits a PCE of 18.32%, which is rare presented in the ternary devices. These achievements demonstrate that our multi‐functional third component design strategies provide insights for high tolerance to ratios and efficiency promotion in multi‐component OPVs, and a feasible way for OPVs’ commercialization.

## Conflict of Interest

The authors declare no conflict of interest.

## Supporting information

Supporting Information

## Data Availability

The data that support the findings of this study are available from the corresponding author upon reasonable request.
