# Supplementary material for: The Multi‐Functional Third Acceptor Realizes the Synergistic Improvement in Photovoltaic Parameters and the High‐Ratio Tolerance of Ternary Organic Photovoltaics
Source: Adv Sci (Weinh). 2024 Aug 13;11(39):2405303. doi: 10.1002/advs.202405303 (PMC11497047; doi:10.1002/advs.202405303)
Supplement: Supplementary file 1 — Supporting Information [file ADVS-11-2405303-s001.docx]

Supporting Information

**The multi-functional third acceptor realizes the synergistic improvement in photovoltaic parameters and the high-ratio tolerance of ternary organic photovoltaics**

*Yuhao Liu, Lingling Zhan,* Zhongjie Li, Hang Jiang, Huayu Qiu, Xiaokang Sun, Hanlin Hu, Rui Sun, Jie Min, Jinyang Yu, Weifei Fu, Shouchun Yin,* and Hongzheng Chen**

Y. Liu, Dr. L. Zhan, Z. Li, H. Jiang, Prof. H. Qiu, Prof. S. Yin

Key Laboratory of Organosilicon Chemistry and Materials Technology of Ministry of Education, College of Materials, Chemistry and Chemical Engineering, Hangzhou Normal University, Hangzhou 311121, P. R. China

E-mail: [linglingzhan@hznu.edu.cn](mailto:linglingzhan@hznu.edu.cn); [yinsc@hznu.edu.cn](mailto:yinsc@hznu.edu.cn)

J. Yu, Dr. W. Fu, Prof. H. Chen

State Key Laboratory of Silicon and Advanced Semiconductor Materials, Department of Polymer Science and Engineering, Zhejiang University, Hangzhou 310027, P. R. China

E-mail: [hzchen@zju.edu.cn](mailto:hzchen@zju.edu.cn)

X. Sun, Prof. H. Hu

Hoffmann Institute of Advanced Materials, Shenzhen Polytechnic University, 518055 Shenzhen, P. R. China

R. Sun, Prof. J. Min

The Institute for Advanced Studies, Wuhan University, Wuhan 430072, P. R. China

**Materials and Methods**

**Instrument.**

**Cyclic voltammetry (CV)** was done on a CHI600A electrochemical workstation with Pt disk, Pt plate, and standard calomel electrode (SCE) as working electrode, counter electrode, and reference electrode, respectively, in a 0.1 mol L^-1^ tetrabutylammoniumhexafluorophosphate (Bu_4_NPF_6_) acetonitrile solution. The CV curves were recorded versus the potential of SCE, which was calibrated by the ferrocene-ferrocenium (Fc/Fc^+^) redox couple (4.8 eV below the vacuum level). The equation of *E*_LUMO/HOMO_ = -e(E_red/ox_+4.41) (eV) was used to calculate the LUMO and HOMO levels (the redox potential of Fc/Fc^+^ is found to be 0.39 V).

**UV-vis absorption spectra** were recorded on a Shimadzu UV-1800 spectrophotometer.

The pumping light wavelength used to excite the samples was 750 nm, with a power of 10 mW. The **photoluminescene spectra** were recorded with an Andor spectrometer (Shamrock sr-303i-B), which was coupled with a Newton electron multiplying CCD detector.

Topographic images of the films were obtained on a VeecoMultiMode **atomic force microscopy (AFM)** in the tapping mode using an etched silicon cantilever at a nominal load of ~2 nN, and the scanning rate for a 10 μm×10 μm image size was 1.5 Hz.

**GIWAXS measurements** were carried out with a Xeuss 2.0 SAXS/WAXS laboratory beamline using a Cu X-ray source (8.05 keV, 1.54 Å) and a Pilatus3R 300K detector. The incidence angle is 0.2^o^.

The structural information of blend films such as the period of arrangement and lamellar stacking spacing is obtained via the Bragg equation. The crystal coherence length (CCL) can be obtained from the Scherrer formula,^1^ and the specific expressions of the Bragg equation and Scherrer formula are as follows:

$$\begin{aligned} d=\frac{\lambda}{2\sin\left( \theta\right)}=\frac{2\pi}{q}\#\left( 1 \right) \end{aligned}$$

$$\begin{aligned} CCL=\frac{K\lambda}{FWHM\cdot\cos\left( \theta\right)}\#\left( 2 \right) \end{aligned}$$

where d is the lamellar stacking spacing, and CCL is the crystal domain along the specified direction called crystal coherence length, which is generally considered to be equivalent to the grain size. 𝜆 is the value of X-ray wavelength; K is a dimensionless shape factor,^[2](#_ENREF_59)^ generally taken as K = 0.89; FWHM is the half-peak width of the scattering peak; θ is the scattering angle.

**The *J*-*V* measurement** was performed via the solar simulator (SS-F5-3A, Enlitech) along with AM 1.5G spectra whose intensity was calibrated by the certified standard silicon solar cell (SRC-2020, Enlitech) at 100 mw cm^-2^. The external quantum efficiency (EQE) data were obtained by using the solar-cell spectral-response measurement system (RE-R, Enlitech).

For femtosecond **transient absorption (fs-TAS) spectroscopy**, the total output from Yb:KGW laser (1030 nm, 220 fs Gaussian fit, 100 kHz, Light Conversion Ltd) was separated into two light beams. One beam was introduced to NOPA (ORPHEUS-N, Light Conversion Ltd) to produce a specific wavelength for the pump beam (here, we use 800 nm), and the other beam was focused onto a YAG plate to generate a white light continuum as a probe beam. The pump and probe overlapped on the sample at a slight angle of less than 10°. The pump fluence is the same for all samples and is low enough to exclude the influence of exciton-exciton annihilation. A linear CCD array collected the transmitted probe light from the sample. Samples were kept in a N_2_ filled cell at room temperature for all TA measurements.

The charge carrier mobilities of the binary and ternary blend films were measured using the **space-charge-limited current (SCLC)** method. Hole-only devices were fabricated in a structure of indium tin oxide (ITO)/PEDOT:PSS/active layer/MoO_3_/Ag. Electron-only devices were fabricated in a structure of ITO/ZnO/active layer/PFN-Br/Al. The device characteristics were extracted by modeling the dark current under forward bias using the SCLC expression described by the Mott-Gurney law:

$J=\frac{9}{8}\varepsilon_{r}\varepsilon_{0}\mu\frac{V^{2}}{L^{3}}$ (3)

Here, ε_r_ ≈ 3 is the average dielectric constant of the blend film, ε_0_ is the permittivity of the free space, *μ* is the carrier mobility, *L* is the thickness of the film, and *V* is the applied voltage.

**FTPS-EQE Measurements** were recorded using a Bruker Vertex 70 Fourier-transform infrared (FTIR) spectrometer, equipped with a quartz tungsten halogen lamp, a quartz beam-splitter, and an external detector option. A low noise current amplifier (Femto DLPCA-200) was used to amplify the photocurrent produced on the illumination of the photovoltaic devices with light modulated by FTIR. The output voltage of the current amplifier was fed back into the external detector port of FTIR. The photocurrent spectrum was collected by FTIR’s software.

**Electroluminescence (EL) quantum efficiency (EQE_EL_)** measurements were performed by applying external voltage sources through the devices from 1V to 4V. A Keithley 2400 SourceMeter was used for supplying voltages and recording injected currents, and a Keithley 485 picoammeter was used for measuring the emitted light intensity.

**Energy Loss analysis** can be quantified as the following formula:

$$E_{loss}=E_{g}-qV_{oc}=\left( E_{g}-qV_{oc}^{SQ} \right)+\left( qV_{oc}^{SQ}-qV_{oc}^{rad} \right)+\left( qV_{oc}^{rad}-qV_{oc} \right)=\left( E_{g}-qV_{oc}^{SQ} \right)+q\Delta V_{oc}^{rad,below gap}+q\Delta V_{oc}^{non-rad}=\Delta E_{1}+\Delta E_{2}+\Delta E_{3} (4)$$

where *E*_g_ is the bandgap, $V_{oc}^{SQ}$ is the maximum *V*_OC_ under the SQ limit, and $V_{oc}^{SQ}$is the *V*_OC_ when only radiative recombination is considered. Δ*E*_1_ is an unavoidable part. Δ*E*_2_ is the part affected by the blends' reorganization energy and energy disorder degree. For Δ*E*_3_, it has a quantitative relationship with the luminescence efficiency of photovoltaic materials., which can be expressed as the following formula:

**Materials.**

All reagents and solvents, unless otherwise specified, were purchased from commercial sources and were used without further purification. D18 and Y6 were purchased from Solarmer Inc. BTP-SA1, BTP-SA2 and BTP-SA3 were synthesized by our group.

**Device Fabrication and Characterization.**

Organic photovoltaics (OPVs) were fabricated on glass substrates commercially pre-coated with a layer of ITO with the conventional structure of ITO/PEDOT:PSS/Active Layer/PDINN/Ag. Prior to fabrication, the substrates were cleaned using detergent, deionized water, acetone and isopropanol consecutively for 10 min in each step, and then treated in an ultraviolet ozone generator for 20 min before being spin-coated at 4000 rpm with a layer of 10 nm thick PEDOT:PSS (Baytron P AI4083). After baking the PEDOT:PSS layer in air at 150 ^o^C for 20 min, the substrates were transferred to a glovebox. The active layer was spin coated from the chloroform solution of 1, 5-dichloro-1-obenzene (DCBB) with 10 mg/mL concentration, at the concentration of 4.5 mg/mL for Donor materials (D:A = 1:1.4) at 3000 rpm for 30 s to around 100 nm. For ternary blends, keep the total D:A ratio as 1:1.4, and Y6:BTP-SA1~3 in the ratio of 0.9:0.1. For LbL-type device, keep the donor concentration of 4.5 mg/mL (pure chloroform solutions), and Y6:BTP-SA1~3 in the ratio of 0.9:0.1 (chloroform solutions of 1, 5-DCBB with 10 mg/mL concentration). An extra pre-annealing at 100 ^o^C for 10 min was performed, and then a 5 nm thick PDINN film was deposited as the cathode buffer layer by the spin-coating of a solution of 1 mg/mL PDINN in methanol. Finally, the Ag (100 nm) electrode was deposited by the thermal evaporation to complete the device with an active area of 6 mm^2^, and the testing aperture area is 4.73 mm^2^.

**Synthesis of BTP-SA1, BTP-SA2, and BTP-SA3**

**BTP-SA1：**

Compound 1 (0.21 g, 0.19 mmol), compound 3 (0.06 g, 0.3 mmol), and compound 4 (0.08 g, 0.3 mmol) in chloroform (40 mL) were added to a Schlenk tube; Freeze with liquid nitrogen, pump three times, thaw; Under the protection of nitrogen, 1 mL of pyridine was injected into the thawed mixing system, and the reactants were reflowed at 65 ℃ for 7 h to produce the Knoevenagel reaction. After the reaction was finished, the black solid BTP-SA1 (0.19 g, 55%) was obtained by column purification (petroleum ether: dichloromethane = 1:1, v/v). ^1^H NMR (500 MHz, CDCl_3_) *δ* = 9.18 (s, 1H), 8.80 (s, 1H), 8.25 (s, 1H), 8.03 (d, 2H), 7.96 (s, 1H), 4.77 (s, 4H), 3.23 (m, 4H), 2.12 (s, 2H), 1.89 (d, 5H), 1.60-1.45 (m, 19H), 1.31 (d, 82H), 1.12-0.74 (m, 60H), 0.67 (m, 15H).

**BTP-SA2：**

Compound 2 (0.22 g, 0.19 mmol), compound 3 (0.06 g, 0.3 mmol), and compound 4 (0.08g, 0.3 mmol) in chloroform (40 mL) were added to a Schlenk tube; Freeze with liquid nitrogen, pump three times, thaw; Under the protection of nitrogen, 1 mL of pyridine was injected into the thawed mixing system, and the reactants were reflowed at 65 ℃ for 7 h to produce the Knoevenagel reaction. After the reaction was finished, the dark brown solid BTP-SA2 (0.2 g, 56%) was obtained by column purification (petroleum ether: dichloromethane = 1:1, v/v). ^1^H NMR (500 MHz, CDCl_3_) *δ* = 9.15 (s, 1H), 8.78 (s, 1H), 8.25 (s, 1H), 8.02 (d, 2H), 7.95 (s, 1H), 4.78 (d, 4H), 3.15 (dd, 5H), 2.16-2.04 (m, 5H), 1.56 (s, 4H), 1.50-1.38 (m, 10H), 1.40-1.30 (m, 12H), 1.25 (m, 20H), 1.11 (m, 11H), 1.07-0.90 (m, 24H), 0.86 (m, 20H), 0.66 (m, 14H).

**BTP-SA3：**

Compound 2 (0.22 g, 0.19 mmol), compound 5 (0.06 g, 0.3 mmol), and compound 4 (0.08 g, 0.3 mmol) in chloroform (40 mL) were added to a Schlenk tube; Freeze with liquid nitrogen, pump three times, thaw; Under nitrogen protection, 1 mL of pyridine was injected into the thawed mixing system. The Knoevenagel reaction occurred at 65 ℃ reflux for 7 h. After the reaction was finished, the dark brown solid BTP-SA3 (0.2 g, 56%) was obtained by column purification (petroleum ether: dichloromethane = 1:1, v/v). ^1^H NMR (500 MHz, CDCl_3_) *δ* = 9.09 (s, 1H), 8.72 (s, 1H), 8.16 (s, 1H), 7.88 (s, 1H), 7.68 (t, 3H), 4.70 (d, 5H), 3.08 (m, 6H), 2.03 (d, 7H), 1.48 (s, 5H), 1.38 (s, 13H), 1.30 (s, 9H), 1.19 (s, 15H), 1.06 (s, 14H), 0.97-0.85 (m, 27H), 0.80 (m, 24H), 0.58 (m, 18H).

**Supplementary Figures**

**Figure S1** ^1^H NMR spectrum of BTP-SA1.

**Figure S2** ^1^H NMR spectrum of BTP-SA2.


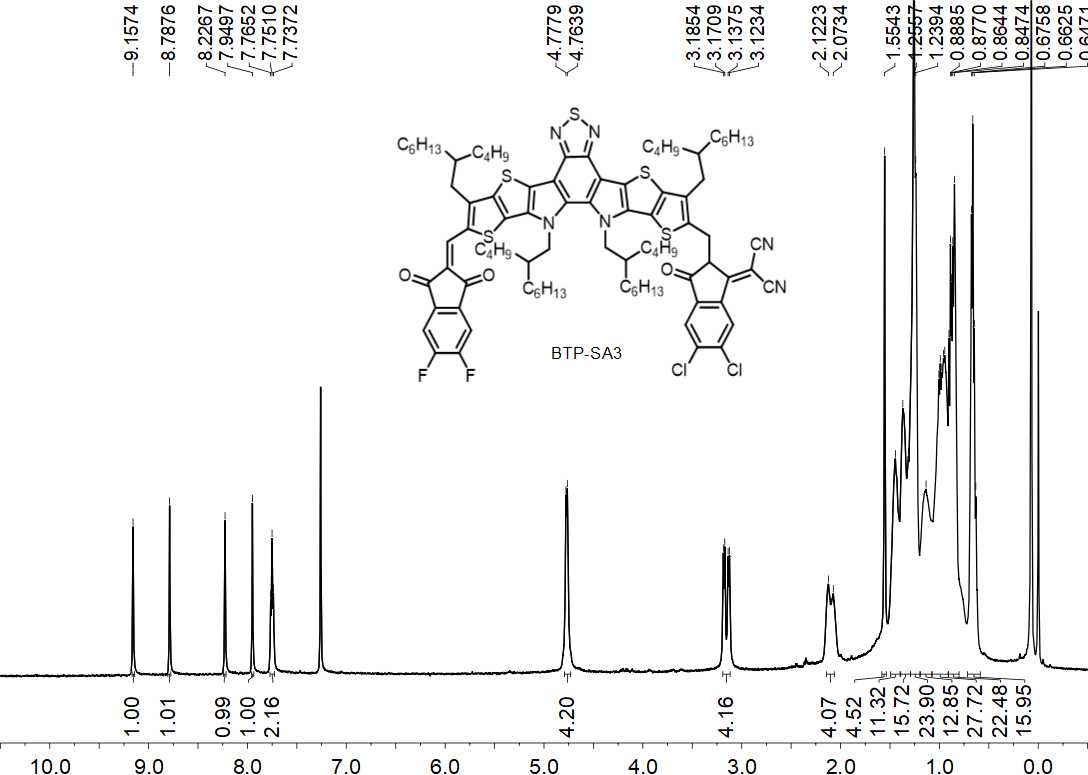


**Figure S3** ^1^H NMR spectrum of BTP-SA3.


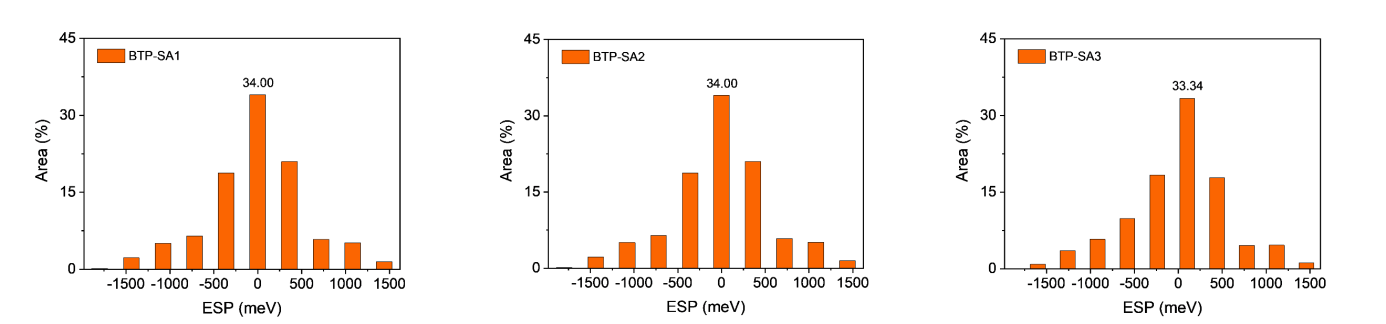


**Figure S4** The histograms of electrostatic potential area distribution of BTP-SA1, BTP-SA2 and BTP-SA3.


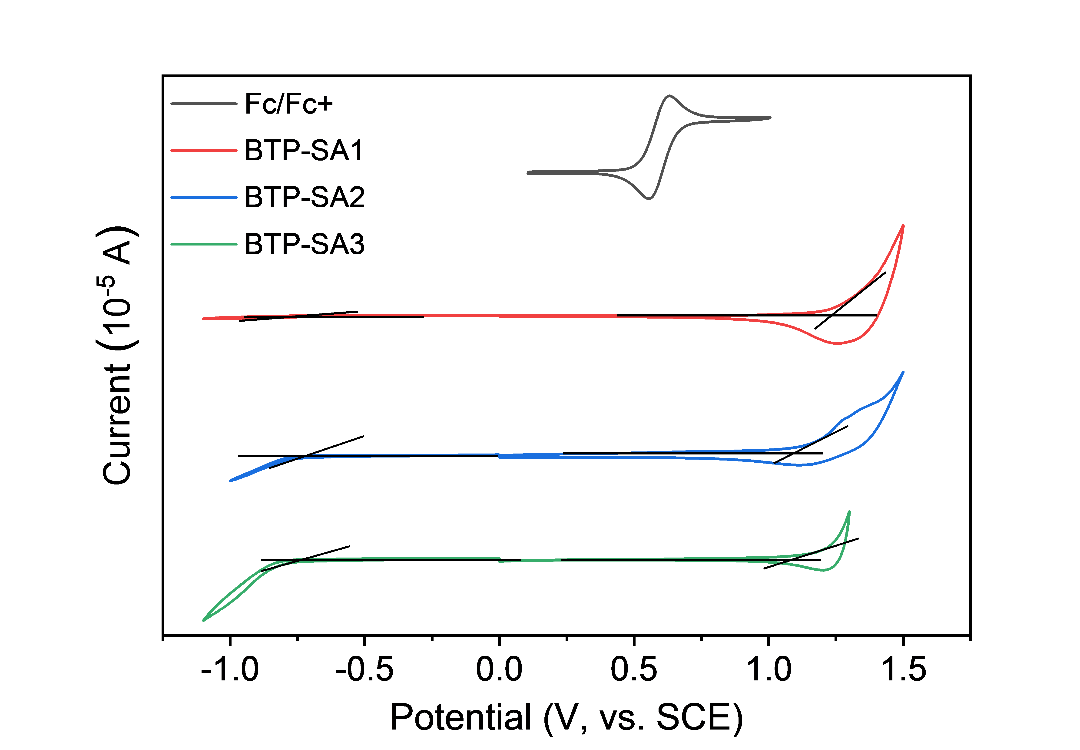


**Figure S5** Cyclic voltammograms of BTP-SA1, BTP-SA2, BTP-SA3 and Fc/Fc+.


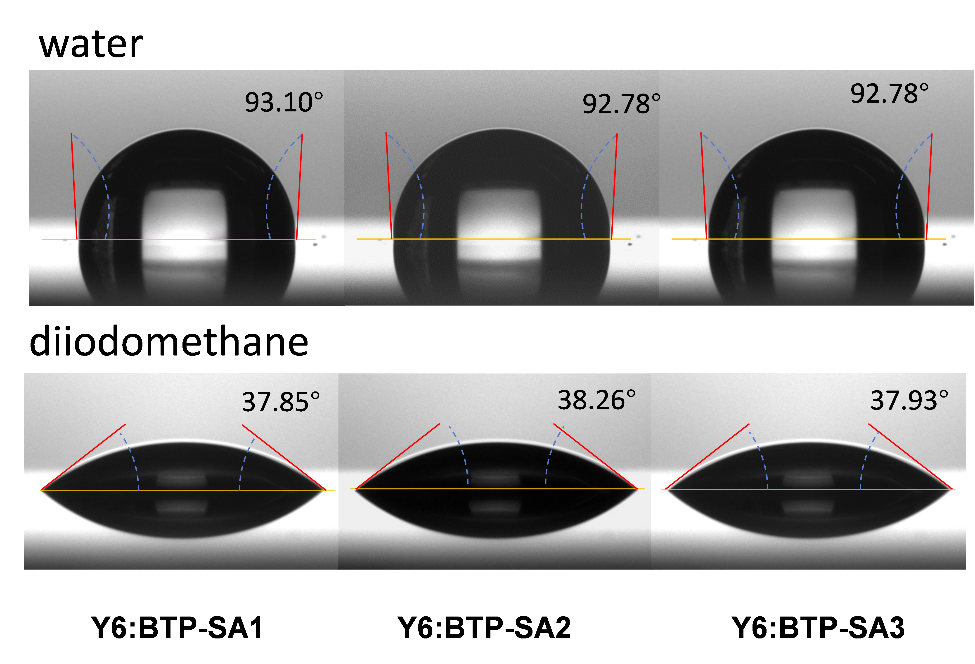


**Figure S6** Contact angle images of various acceptor-mixed films with water and diiodomethane droplets on top.


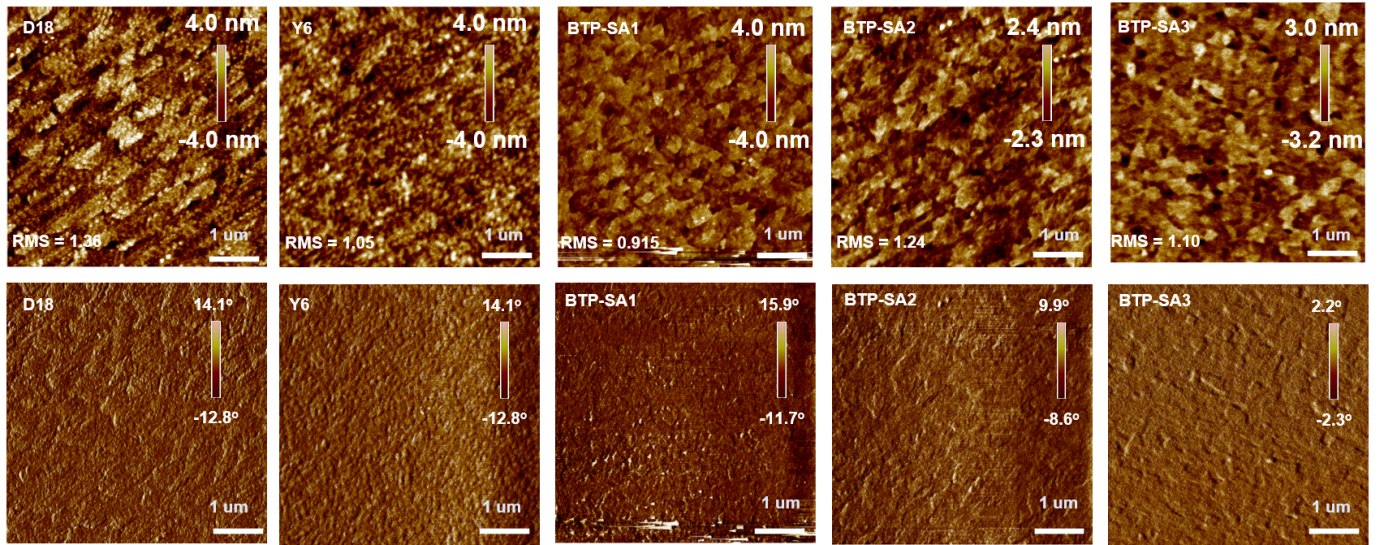


**Figure S7** AFM height and phase images of various neat films.

**Figure S8** Phase images of various blended films.


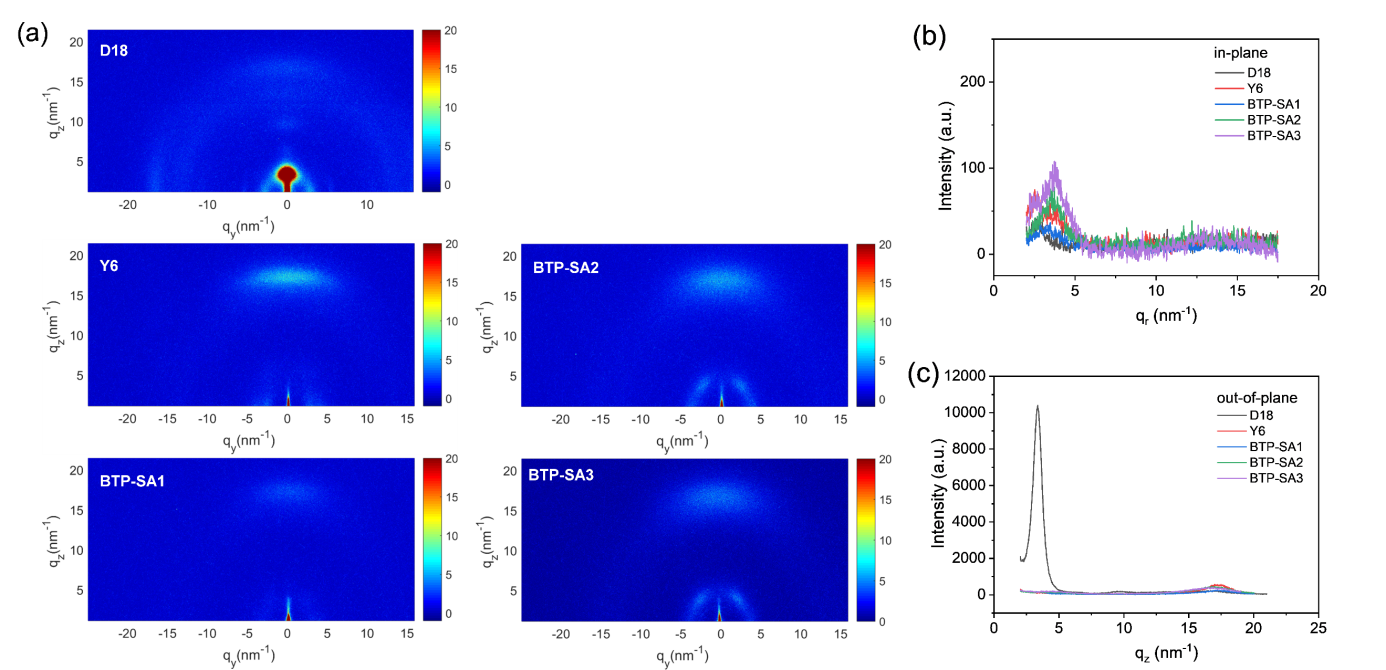


**Figure S9** a) 2D GIWAXS images of the neat films. b) The in-plane and c) out-of-plane directions.


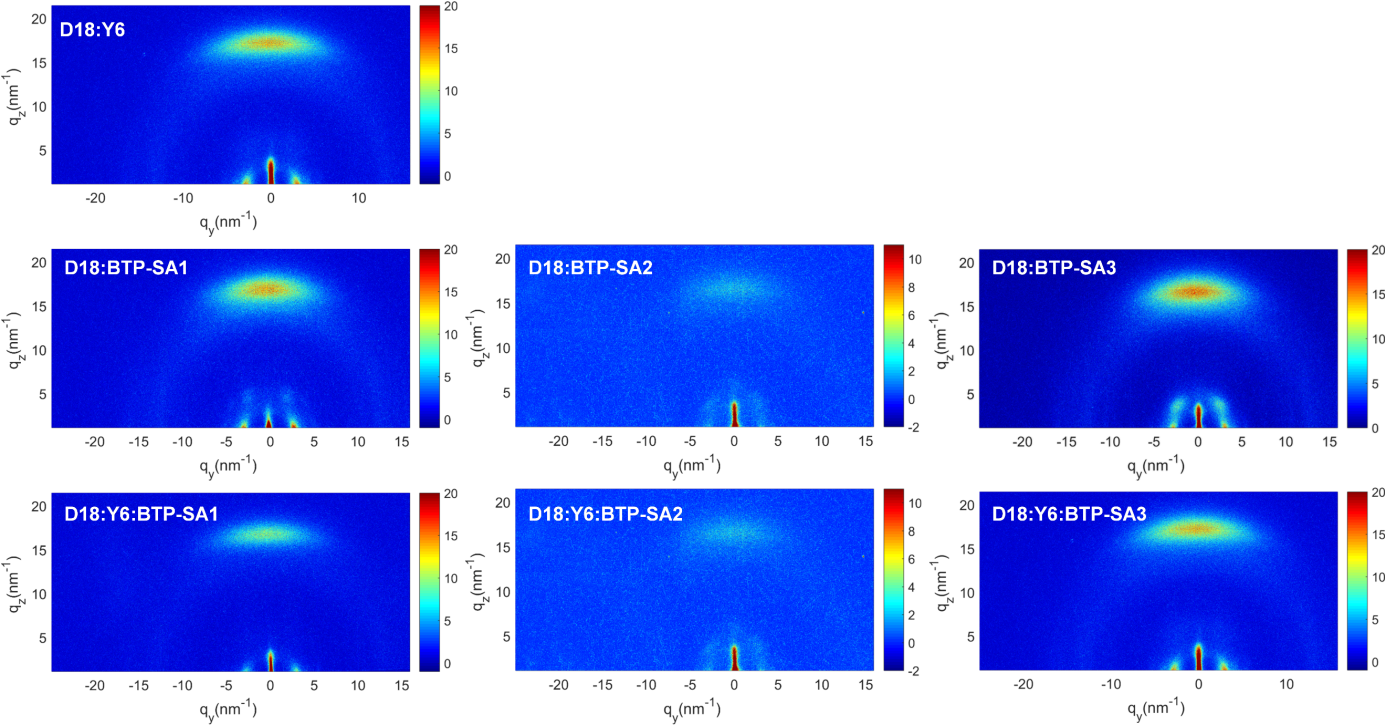


**Figure S10** 2D GIWAXS images of the various blended films.

**
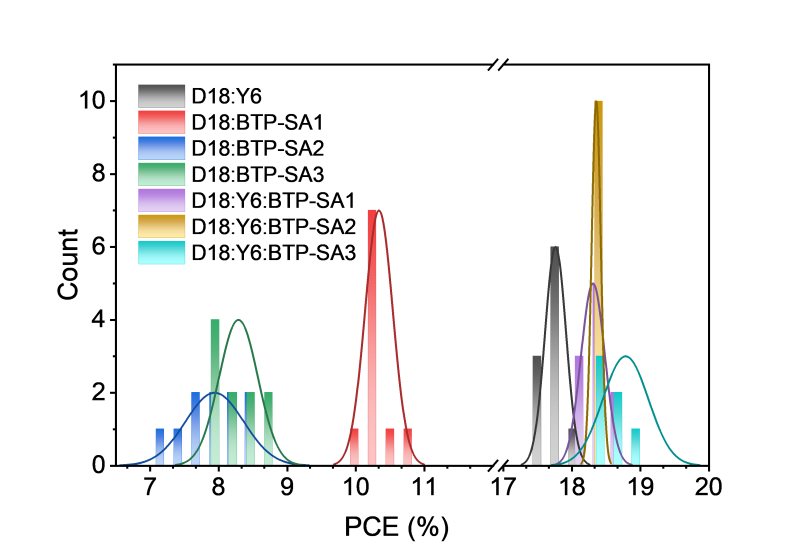
**

**Figure S11** Histograms of PCE values of various binary and ternary devices.


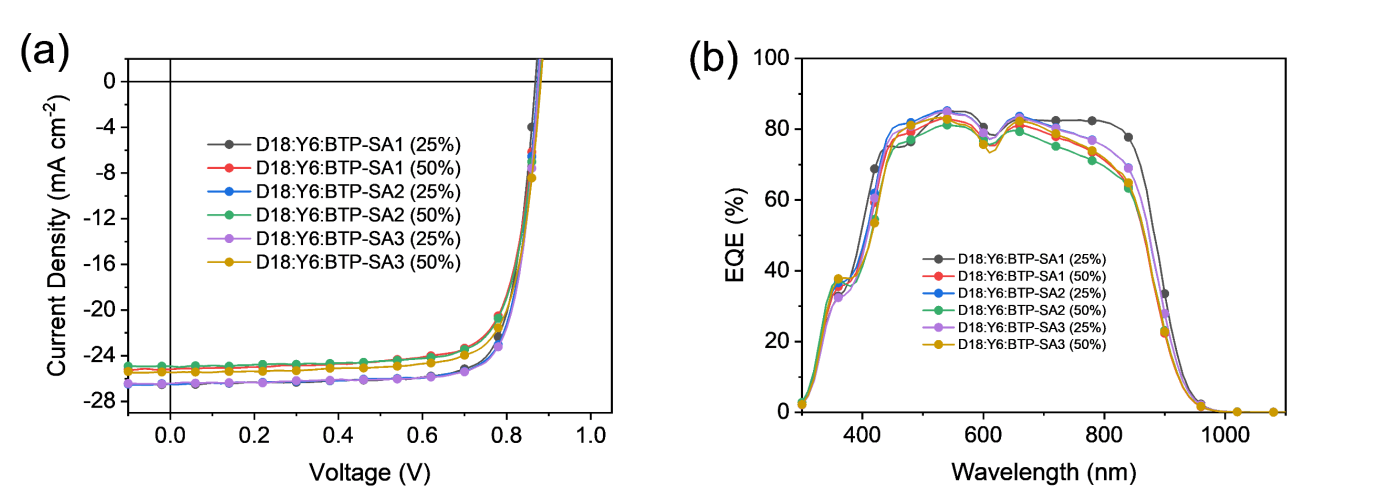


**Figure S12** a) *J*-*V* curve, and b) EQE curve of BHJ-type device based on three ternary systems (D18:Y6:BTP-SA1, D18:Y6:BTP-SA2, and D18:Y6:BTP-SA3) with various addition ratio.


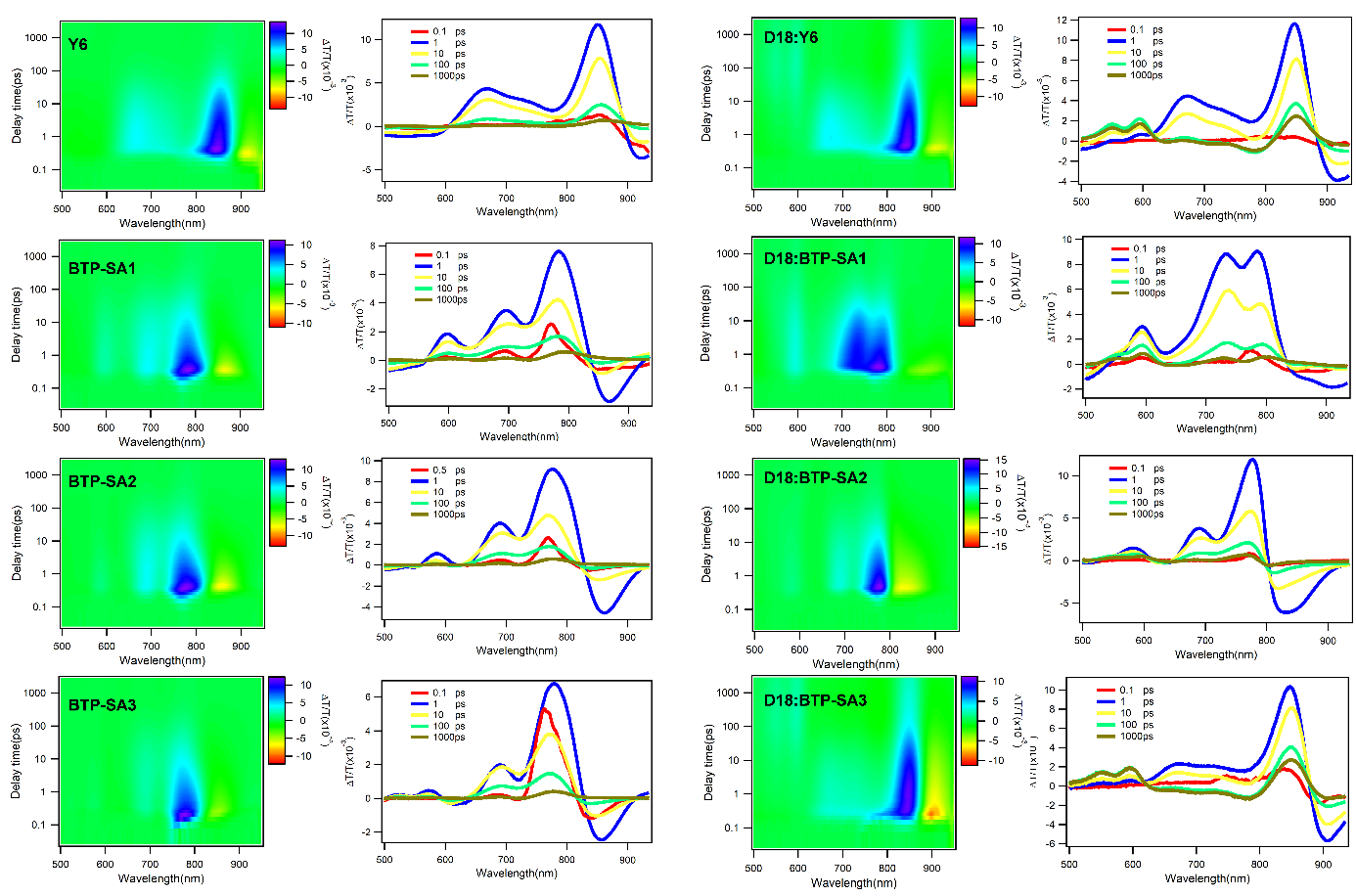


**Figure S13** Color plot of TA spectra of neat films and different binary blends under 800 nm excitation.


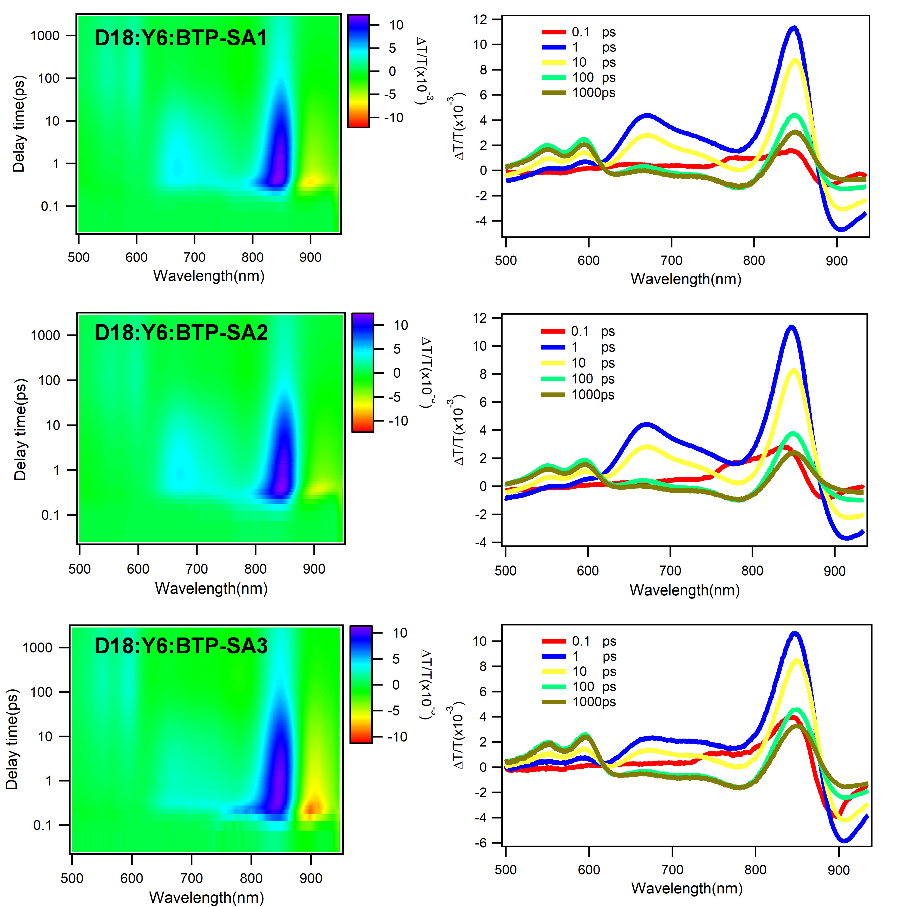


**Figure S14** Color plot of TA spectra of different ternary blends under 800 nm excitation.


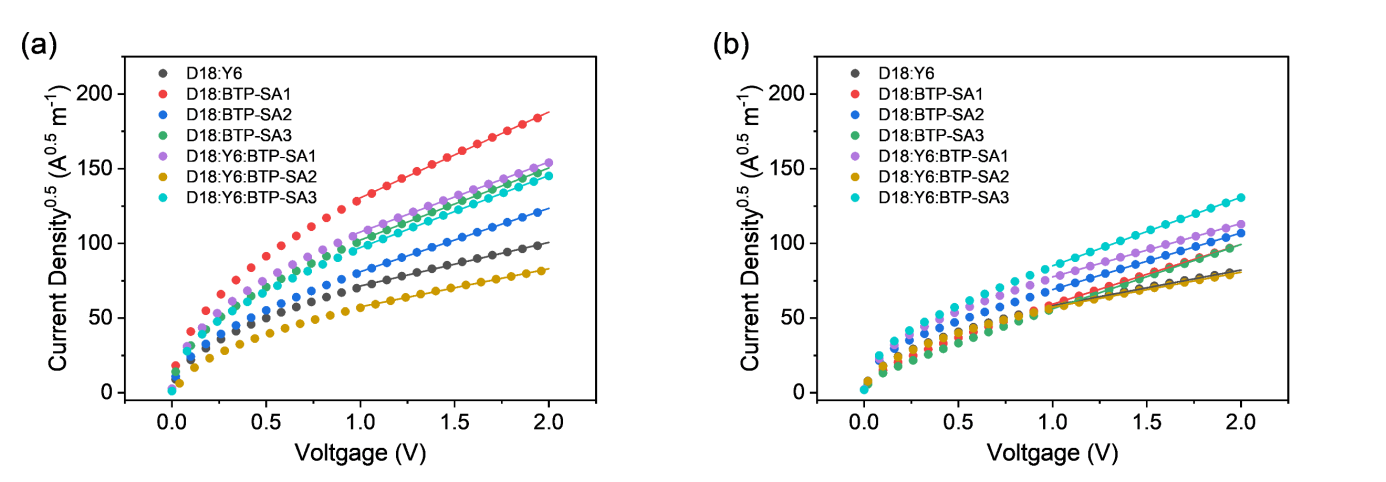


**Figure S15** a) *J*^0.5^-*V* curves of the hole-only BHJ-type devices. b) *J*^0.5^-*V* curves of the electron-only BHJ-type devices.


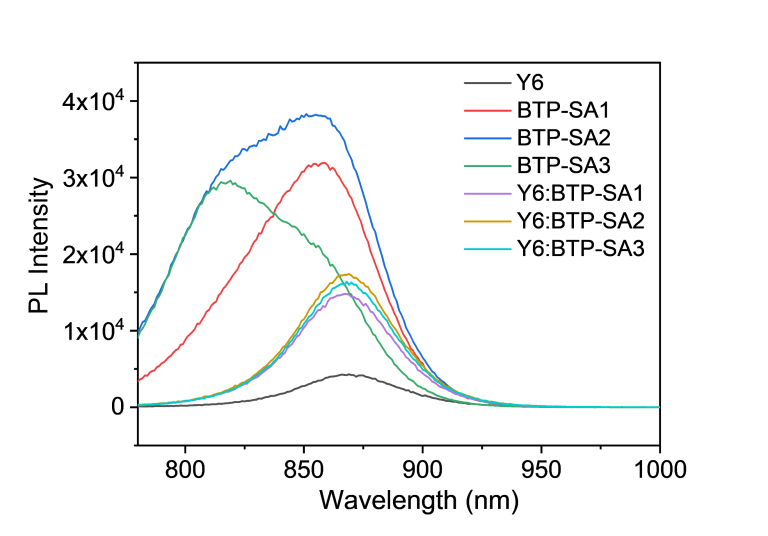


**Figure S16** The fluorescence spectra of various acceptors.


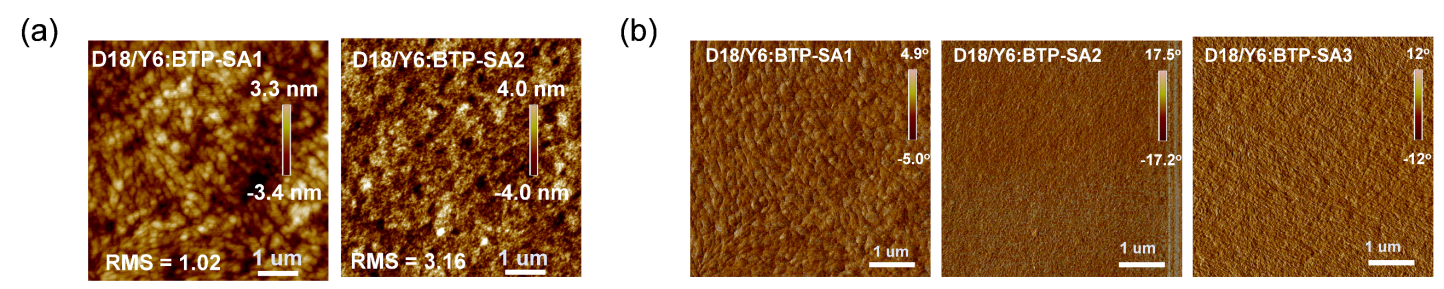


**Figure S17** a) AFM height images of D18/Y6:NBTP-SA1 and D18/Y6:NBTP-SA2. b) Phase images of various LbL-type ternary films.


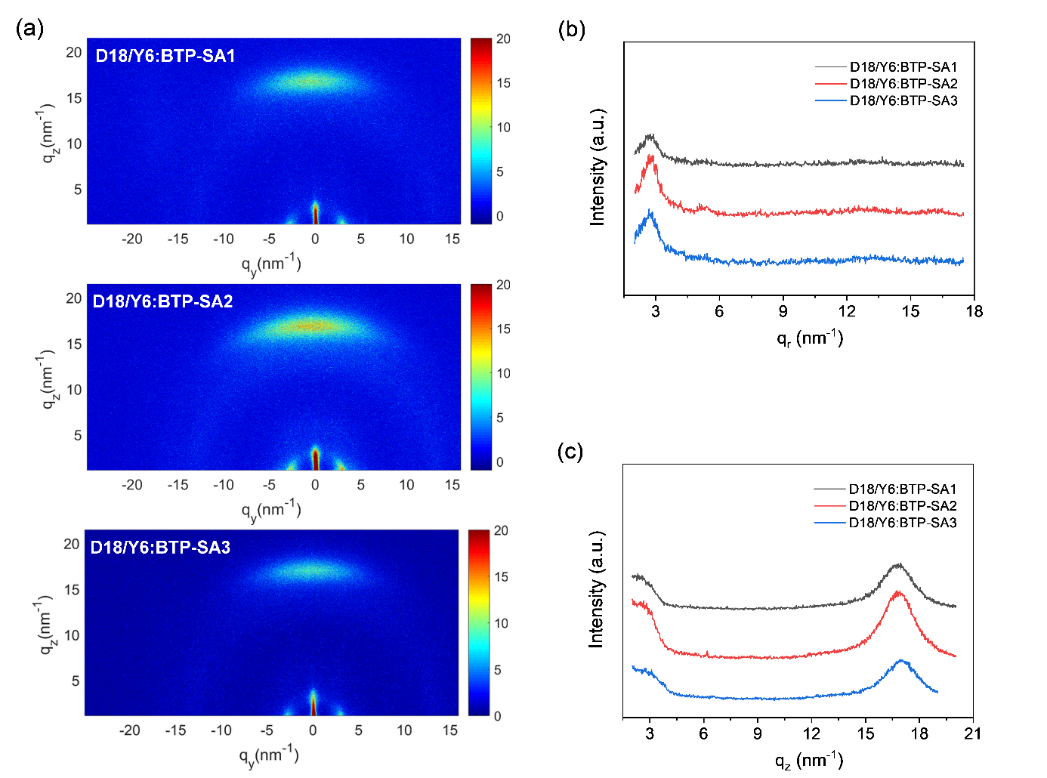


**Figure S18** a) 2D GIWAXS images, b) the in-plane, and c) out-of-plane directions of the various LbL-type ternary films.


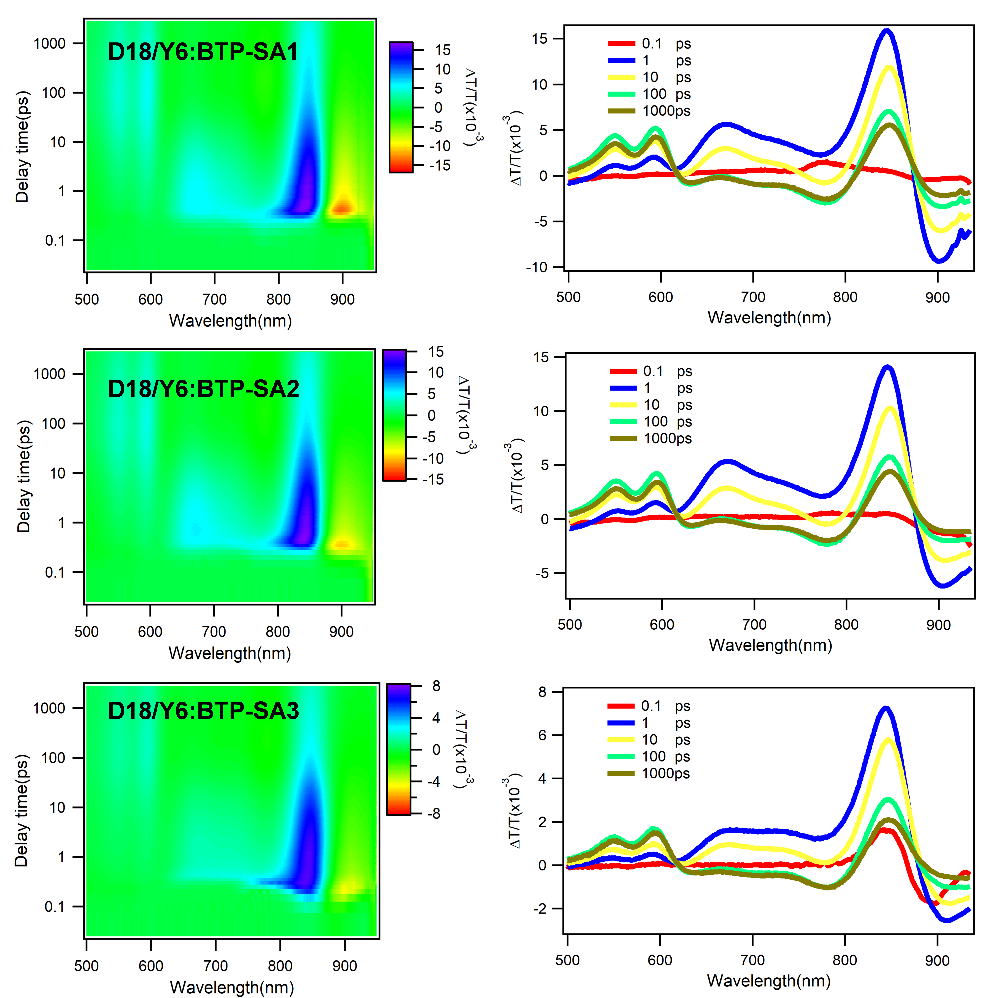


**Figure S19** Color plot of TA spectra of different LbL-type ternary blends under 800 nm excitation.


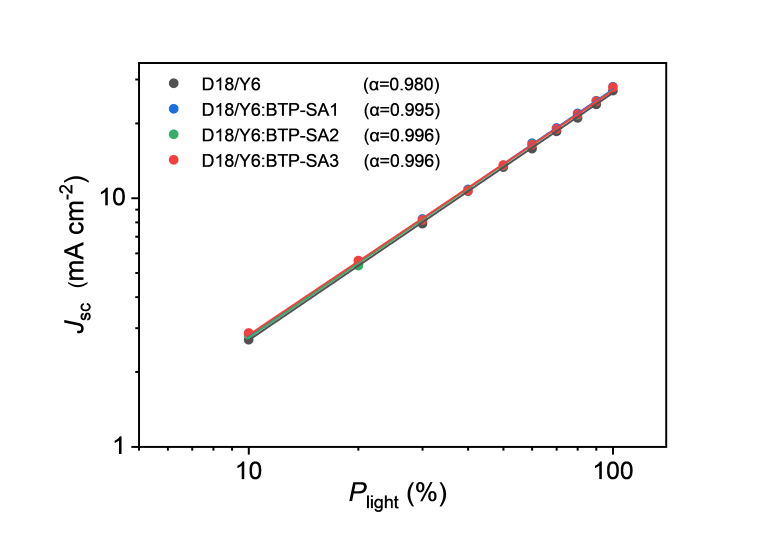


**Figure S20** Dependence of *J*_SC_ on light intensity of the LbL-type ternary devices.


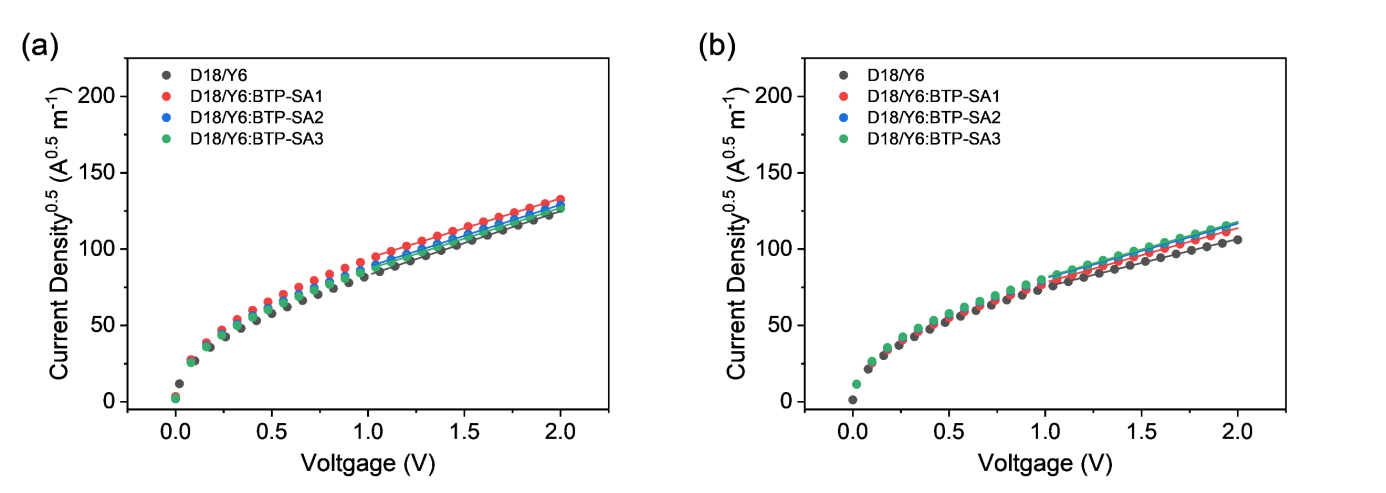


**Figure S21** a) *J*^0.5^-*V* curves of the hole-only LbL-type devices. b) *J*^0.5^-*V* curves of the electron-only LbL-type devices.


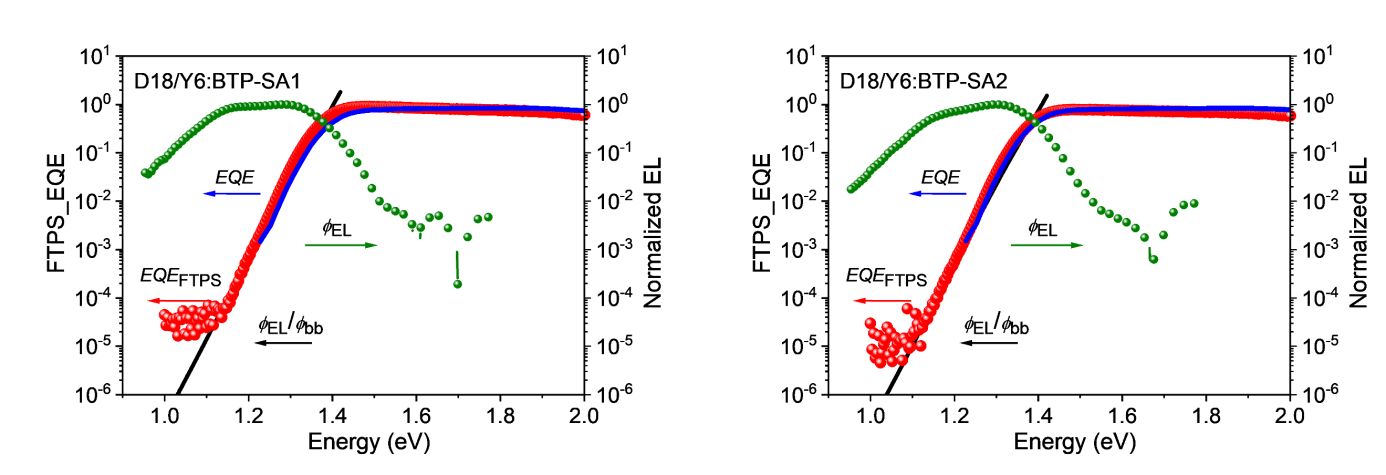


**Figure S22** Semilogarithmic plots of normalized EL spectra, measured EQE spectra, and FTPS-EQE spectra as a function of energy for devices based on D18/Y6:BTP-SA1 and D18/Y6:BTP-SA2 devices.

**Supplementary Tables**

**Table S1** Summary of dispersion (*γ^d^*) and polar (*γ^p^*) components of surface tensions, surface tensions (*γ*), Flory-Huggins interaction parameters (*χ*), and wetting coefficient (*ω*) for various films.

| Surface | $\gamma^{d}$  (mN m^-1^) | $\gamma^{p}$  (mN m^-1^) | γ  (mN m^-1^) | $\chi^{D-A}$^a^ | $\gamma_{D/A}$^b^ | $\gamma_{A_{2}/A}$ | | $\omega_{A_{2}}$ *^c^* |
| --- | --- | --- | --- | --- | --- | --- | --- | --- |
| D18 | 34.66 | 0.04 | 34.70 | / |  |  |  |  |
| Y6 | 40.84 | 0.32 | 41.16 | / | / | / | / | / |
| BTP-SA1 | 43.35 | 0.09 | 43.44 | / | / | / | / | / |
| BTP-SA2 | 42.41 | 0.07 | 42.49 | / | / | / | / | / |
| BTP-SA3 | 41.54 | 0.15 | 41.69 | / | / | / | / | / |
| Y6:BTP-SA1 | 40.79 | 0.42 | 41.20 | / | / | γ_Y6/SA1_ | 0.20 | -1.09 |
| Y6:BTP-SA2 | 40.46 | 0.48 | 40.93 | / | / | γ_Y6/SA2_ | 0.18 | -0.84 |
| Y6:BTP-SA3 | 40.53 | 0.52 | 41.06 | / | / | γ_Y6/SA3_ | 0.07 | -0.86 |
| D18:Y6 | / | / | / | 0.28 | 0.72 | / | / | / |
| D18:BTP-SA1 | / | / | / | 0.49 | 0.99 | / | / | / |
| D18:BTP-SA2 | / | / | / | 0.39 | 0.79 | / | / | / |
| D18:BTP-SA3 | / | / | / | 0.32 | 0.68 | / | / | / |
| D18:Y6:BTP-SA1 | / | / | / | 0.28 | 0.81 | / | / | / |
| D18:Y6:BTP-SA2 | / | / | / | 0.26 | 0.82 | / | / | / |
| D18:Y6:BTP-SA3 | / | / | / | 0.27 | 0.87 | / | / | / |

^a^ The Flory-Huggins interaction parameter between the donor (D) and acceptor (A) is calculated through the equation of $\chi^{D-A}={(\sqrt{\gamma_{D}}-\sqrt{\gamma_{A}})}^{2}$.

^b^ The interfacial tension between A and B can be calculated through the Wu’s Equation of $\gamma_{A/B}= \gamma_{A}+ \gamma_{B}-4\left( \frac{{\gamma_{A}}^{d}{\gamma_{B}}^{d}}{{\gamma_{A}}^{d}+{\gamma_{B}}^{d}}+\frac{{\gamma_{A}}^{p}{\gamma_{B}}^{p}}{{\gamma_{A}}^{p}+{\gamma_{B}}^{p}} \right)$. *^c^* The wetting coefficient (*ω*) of the third component A_2_ in the mixture of D:A_1_ can be expressed according to Young's equation of $\omega_{A_{2}}=\frac{\gamma_{A_{1}{/A}_{2}}-\gamma_{{D/A}_{2}}}{\gamma_{D/A_{1}}}$.

| Film | Position (nm^-1^) | FWHM (nm^-1^) | D-Spacing (nm) | CCL (nm) |
| --- | --- | --- | --- | --- |
| D18:Y6 | 17.24 | 2.319 | 0.36 | 4.85 |
| D18:BTP-SA1 | 16.92 | 2.177 | 0.37 | 5.16 |
| D18:BTP-SA2 | 16.66 | 2.209 | 0.38 | 5.09 |
| D18:BTP-SA3 | 16.63 | 1.908 | 0.38 | 5.89 |
| D18:Y6:BTP-SA1 | 16.97 | 1.807 | 0.37 | 6.22 |
| D18:Y6:BTP-SA2 | 16.87 | 1.828 | 0.37 | 6.15 |
| D18:Y6:BTP-SA3 | 17.22 | 1.711 | 0.36 | 6.57 |

**Table S2** Structure parameters of the various neat films, BHJ-type binary and ternary blended films obtained from GIWAXS data.

**Table S3** The photovoltaic parameters of the devices based on three ternary systems (D18:Y6:BTP-SA1, D18:Y6:BTP-SA2, and D18:Y6:BTP-SA3) with various addition ratio.

| Active Layer | *V*_OC_  (V) | *J*_SC_  (mA cm*^-2^*) | *J*_cal_ *^a^*  (mA cm*^-2^*) | FF  (%) | PCE *^b^*  (%) |
| --- | --- | --- | --- | --- | --- |
| D18:Y6:BTP-SA1 (wt%:25%) | 0.868  (0.866 ± 0.003) | 26.45  (26.39 ± 0.37) | 26.18 | 78.78  (77.37 ± 1.47) | 18.09  (17.69 ± 0.27) |
| D18:Y6:BTP-SA1 (wt%:50%) | 0.876  (0.875 ± 0.002) | 25.03  (24.25 ± 0.40) | 24.32 | 75.33  (74.95 ± 0.81) | 16.62  (16.19 ± 0.52) |
| D18:Y6:BTP-SA2 (wt%:25%) | 0.873  (0.872 ± 0.005) | 26.49  (26.43 ± 0.28) | 25.44 | 79.03  (78.64 ± 0.46) | 18.29  (18.13 ± 0.48) |
| D18:Y6:BTP-SA2 (wt%:50%) | 0.879  (0.881± 0.002) | 24.91  (23.78 ± 0.87) | 23.83 | 76.58  (75.52 ± 1.02) | 16.74  (15.80 ± 0.58) |
| D18:Y6:BTP-SA3 (wt%:25%) | 0.875  (0.872 ± 0.003) | 26.41  (26.52 ± 0.28) | 25.28 | 79.68  (79.11 ± 0.57) | 18.43  (18.30 ± 0.13) |
| D18:Y6:BTP-SA3 (wt%:50%) | 0.881  (0.882 ± 0.003) | 25.45  (24.69 ± 0.40) | 24.40 | 76.84  (76.33 ± 0.79) | 17.20  (16.60 ± 0.30) |

**Table S4** The Comparisons of τ_1_ and τ_2_ of different BHJ blends.

| Films | τ_1_ (ps) | A1 (%) | τ_2_ (ps) | A2 (%) |
| --- | --- | --- | --- | --- |
| D18:Y6 | 0.791 ± 0.019 | 39.63 | 17.954 ± 0.254 | 60.37 |
| D18:BTP-SA1 | 0.615 ± 0.051 | 74.72 | 3.543 ± 0.775 | 25.28 |
| D18:BTP-SA2 | 0.327 ± 0.018 | 82.37 | 8.161 ± 1.650 | 17.63 |
| D18:BTP-SA3 | 0.648 ± 0.031 | 32.27 | 30.796 ± 0.553 | 67.73 |
| D18:Y6:BTP-SA1 | 0.813 ± 0.027 | 31.83 | 23.044 ± 0.328 | 68.17 |
| D18:Y6:BTP-SA2 | 0.881 ± 0.037 | 28.71 | 22.562 ± 0.343 | 71.29 |
| D18:Y6:BTP-SA3 | 0.569 ± 0.028 | 34.71 | 24.913 ± 0.515 | 65.29 |

**Table S5** Hole and electron mobilities of BHJ-type binary and ternary blended films.

| Sample | *μ*_h_ (×10^-4^ cm^2^V^-1^s^-1^) | *μ*_e_ (×10^-4^ cm^2^V^-1^s^-1^) | *μ*_h_/*μ*_e_ |
| --- | --- | --- | --- |
| D18:Y6 | 6.04 ± 0.02 | 3.81 ± 0.01 | 1.58 |
| D18:BTP-SA1 | 11.04 ± 0.31 | 5.39 ± 0.08 | 2.04 |
| D18:BTP-SA2 | 5.99 ± 0.05 | 3.64 ± 0.04 | 1.64 |
| D18:BTP-SA3 | 7.77 ± 0.06 | 6.20 ± 0.11 | 1.25 |
| D18:Y6:BTP-SA1 | 7.31 ± 0.01 | 4.28 ± 0.01 | 1.70 |
| D18:Y6:BTP-SA2 | 4.98 ± 0.01 | 3.62 ± 0.01 | 1.37 |
| D18:Y6:BTP-SA3 | 7.79 ± 0.12 | 7.00 ± 0.03 | 1.11 |

**Table S6** Structure parameters of various LbL-type ternary blended films obtained from GIWAXS data.

| Film | D-Spacing (nm) | CCL (nm) |
| --- | --- | --- |
| D18/Y6:BTP-SA1 | 0.37 | 5.81 |
| D18/Y6:BTP-SA2 | 0.37 | 5.68 |
| D18/Y6:BTP-SA3 | 0.37 | 6.57 |

**Table S7** The Comparisons of τ_1_ and τ_2_ of different LbL blends.

| Films | τ_1_ (ps) | A1 (%) | τ_2_ (ps) | A2 (%) |
| --- | --- | --- | --- | --- |
| D18/Y6: BTP-SA1 | 0.580 ± 0.015 | 40.21 | 15.226 ± 0.216 | 59.79 |
| D18/Y6: BTP-SA2 | 0.624 ± 0.016 | 41.39 | 14.964 ± 0.227 | 58.61 |
| D18/Y6: BTP-SA3 | 0.553 ± 0.020 | 34.05 | 24.279 ± 0.361 | 65.95 |

**Table S8** Hole and electron mobilities of LbL-type binary and ternary films.

| Sample | *μ*_h_(×10^-4^cm^2^V^-1^s^-1^) | *μ*_e_(×10^-4^cm^2^V^-1^s^-1^) | *μ*_h_/*μ*_e_ |
| --- | --- | --- | --- |
| D18/Y6 | 5.88 ± 0.01 | 3.33 ± 0.01 | 1.76 |
| D18/Y6:BTP-SA1 | 5.15 ± 0.01 | 4.25 ± 0.01 | 1.21 |
| D18/Y6:BTP-SA2 | 5.53 ± 0.01 | 4.32 ± 0.05 | 1.28 |
| D18/Y6:BTP-SA3 | 5.41 ± 0.01 | 4.44 ± 0.01 | 1.21 |

Table S9 Detailed energy losses of OPVs based on LbL-type ternary devices.

| Active layers | *E*_loss_  (eV) | ${\Delta E}_{1}$=$E_{gap}-qV_{oc}^{SQ}$  (eV) | ${\Delta E}_{2}$=$q{\Delta V}_{oc}^{rad}$  (eV) | ${\Delta E}_{3}$=$q{\Delta V}_{oc}^{non-rad}$ (eV) | EQE_EL_  (%) | $\text{Exp. }q\text{∆}V_{oc}^{non-rad}$  $\text{(}\text{eV}\text{)}$ |
| --- | --- | --- | --- | --- | --- | --- |
| D18/Y6:BTP-SA1 | 0.527 | 0.253 | 0.082 | 0.191 | 5.55E-2 | 0.193 |
| D18/Y6:BTP-SA2 | 0.524 | 0.253 | 0.081 | 0.190 | 5.82E-2 | 0.192 |
| D18/Y6:BTP-SA3 | 0.525 | 0.251 | 0.084 | 0.189 | 5.94E-2 | 0.191 |

**References:**

1. A. Mahmood, J. L. Wang, *Sol. RRL* 2020, **4**, 2000337.

2. D.-M. Smilgies, *J. Appl. Crystallogr.* 2009, **42**, 1030.
